# Identification and Validation of Reference Genes for RT-qPCR Analysis in Non-Heading Chinese Cabbage Flowers

**DOI:** 10.3389/fpls.2016.00811

**Published:** 2016-06-10

**Authors:** Cheng Wang, Hong-Mi Cui, Tian-Hong Huang, Tong-Kun Liu, Xi-Lin Hou, Ying Li

**Affiliations:** ^1^State Key Laboratory of Crop Genetics and Germplasm Enhancement, Nanjing Agricultural UniversityNanjing, China; ^2^Key Laboratory of Biology and Germplasm Enhancement of Horticultural Crops in East China, College of Horticulture, Nanjing Agricultural UniversityNanjing, China

**Keywords:** RT-qPCR, *Brassica rapa*, flowers, reference genes, self-incompatibility, geNorm, NormFinder

## Abstract

Non-heading Chinese cabbage (*Brassica rapa* ssp. *chinensis* Makino) is an important vegetable member of *Brassica rapa* crops. It exhibits a typical sporophytic self-incompatibility (SI) system and is an ideal model plant to explore the mechanism of SI. Gene expression research are frequently used to unravel the complex genetic mechanism and in such studies appropriate reference selection is vital. Validation of reference genes have neither been conducted in *Brassica rapa* flowers nor in SI trait. In this study, 13 candidate reference genes were selected and examined systematically in 96 non-heading Chinese cabbage flower samples that represent four strategic groups in compatible and self-incompatible lines of non-heading Chinese cabbage. Two RT-qPCR analysis software, geNorm and NormFinder, were used to evaluate the expression stability of these genes systematically. Results revealed that best-ranked references genes should be selected according to specific sample subsets. *DNAJ, UKN1*, and *PP2A* were identified as the most stable reference genes among all samples. Moreover, our research further revealed that the widely used reference genes, *CYP* and *ACP*, were the least suitable reference genes in most non-heading Chinese cabbage flower sample sets. To further validate the suitability of the reference genes identified in this study, the expression level of *SRK* and *Exo70A1* genes which play important roles in regulating interaction between pollen and stigma were studied. Our study presented the first systematic study of reference gene(s) selection for SI study and provided guidelines to obtain more accurate RT-qPCR results in non-heading Chinese cabbage.

## Introduction

Quantification of mRNA transcript levels analysis is increasingly important in furthering our insight into complex metabolic pathways and signaling networks which underlie physiological and developmental processes. Quantitative reverse transcription-PCR (RT-qPCR) has been used as the main analysis technique to quantify mRNA transcript levels as well as validate high-throughput data due to its high sensitivity, accuracy and specificity in various fields of biological research (Bustin, 2002; Artico et al., 2010). However, the accuracy of RT-qPCR is easily affected by various factors, such as the quality of mRNA samples, enzymatic efficiency in cDNA synthesis, the efficiency of amplification and the differences in transcriptional activity between the tissues or cells analyzed (Pfaffl, 2001; Derveaux et al., 2010). To avoid bias in RT-qPCR analysis, reliable internal controls, termed reference genes that are steadily expressed in different experimental condition, are essential for normalization.

Some of the commonly used reference genes in plants, such as beta-tubulin-4 (*TUB4*), polyubiquitin (*UBQ*), glyceraldehyde-3-phosphate dehydrogenase (*GAPDH*), 18S ribosomal RNA (*18S rRNA*), actin 7 (*ACT7*), DNAJ-like protein (*DNAJ*), and alpha tubulin-5 (*TUA5*), mostly involving in basic cellular processes, such as intermediary metabolism and protein translation and thus, have been widely adopted for gene expression analyses in different plant species (Die et al., 2010; Kozera and Rapacz, 2013; Wei et al., 2013; Xiao et al., 2014; Delporte et al., 2015). In addition, some new reference genes including F-box protein (*F-box*), acyl carrier proteins (*ACP*), phosphoenolpyruvate carboxylase-related kinase 1 (*PEPKR1*), hypothetical proteins of unknown function (*UNK1*), SAND family protein (*SAND*), and TAP42-interacting protein of 41 kDa (*TIP41*), were identified and found to express stably (Libault et al., 2008; Chang et al., 2012; Nakashima et al., 2013). However, no single reference gene can always keep its stability under variable conditions (Kim et al., 2003; Argyropoulos et al., 2006; Fu et al., 2013; Niu et al., 2015). A systematic validation of the expression stability of candidate reference genes in each experimental system should be carried out before using these reference genes for normalization data in gene expression analysis. Meanwhile, several statistical algorithms, such as geNorm (Vandesompele et al., 2002), NormFinder (Andersen et al., 2004) and BestKeeper (Pfaffl et al., 2004), were well developed to facilitate the evaluation of potential reference gene(s) expression stability from a set of candidate genes under different experimental conditions.

Self-incompatibility is a genetic system in flowering plants that prevents self-fertilization and encourages outcrossing to generate genetic diversity (De Nettancourt, 2001; Watanabe et al., 2001; Indriolo et al., 2014). Non-heading Chinese cabbage (*Brassica rapa* ssp. *chinensis* Makino), an important leaf vegetable cultivated and consumed in Asia, exhibits a typical sporophytic SI system (Wang et al., 2014a). The sporophytic SI trait widely exists in numerous Brassica crops, including Chinese cabbage, radish, broccoli and rape. It has been used as an important agronomic trait for efficient hybrid seed production of these crops. Viewed from another perspective, the self/non-self discrimination in SI between pollen and stigma is an excellent model system for intercellular communication and signal transduction in higher plants (Watanabe et al., 2012). However, the underlying mechanisms explaining this biological process is still not well understood. Thus, exploring the expression patterns of key genes associated with SI of Brassica crops will not only help us to understand the SI mechanism but also promote molecular breeding in *Brassica species* to ease the difficulty of year-round seed production. Some studies have been conducted to select appropriate reference genes in *Brassica* species, such as *Brassica napus* (Chen et al., 2010), *Brassica juncea* (Chandna et al., 2012) and Chinese cabbage (Qi et al., 2010; Xiao et al., 2012). However, all above reference gene studies were about development and stress. Nevertheless, little information, if any, is available concerning reference genes for SI study. Hence, potential reference gene should be defined before assessing the expression of target genes in SI plant.

In previous studies of SI, the molecular events associated with floral development, stigma development, various floral tissues, and pollen-stigma interaction were often used to understand SI in Brassicaceae family (Nasrallah et al., 2002; Loraine et al., 2013; Indriolo et al., 2014; Matsuda et al., 2014; Zhang et al., 2015). Furthermore, there were evidences that thousands of genes changed their expression levels during above events. Thus, the genes expressed of flowers under these experimental conditions need an in-depth study. In this study, the expression stability of 13 candidate reference genes (*ACT7, EF1*α, *TUB4, GAPDH, CYP, DNAJ, HIS, TUA5, ACP, UKN1, SKIP16, CAC*, and *PP2A*) was validated by RT-qPCR across a diverse set of flower samples representing different experimental conditions including different flower developmental stages, different stigma developmental stages, various floral tissues, and stigmas after self-pollination in SI and SC lines of non-heading Chinese cabbage. These genes either stably expressed in *Brassica* species according to previous studies, such as elongation factor-1α (*EF1a*) (Qi et al., 2010; Xiao et al., 2012), cyclophilin (*CYP*) (Qi et al., 2010), clathrin adaptor complex (*CAC*) (Chandna et al., 2012), or have been proved to perform well in flowers of other species, like histone H3 (*HIS*) (Imai et al., 2014), protein phosphatase 2A (*PP2A*) (Jin et al., 2013), SKP1/Ask-interacting protein 16 (*SKIP16*) (Fu et al., 2013). The statistical software geNorm and NormFinder were used to identify the most stable reference genes needed for normalization. Based on the statistical result, expression analysis of two genes of interest related to pollen-stigma recognition, S-locus receptor kinase (*SRK*) and *Exo70A1* during SI response, were presented. Our study is the first analysis about validation of reference genes in flowers of non-heading Chinese cabbage and sets a precedent for future similar study in other species.

## Materials and methods

### Plant material

The self-incompatible line “NHCC 002”and compatible line “NHCC 210” of non-heading Chinese cabbage used in this research were provided by Professor Xilin Hou. The incompatible line is a stable inbred line with a low (<1.0) self-compatibility index (Cao et al., 2002), whereas that of the compatible line is higher than 5.0. Seeds were sowed in October 2014, grown in pots containing a soil: vermiculite (3:1) mixture in a controlled-environment growth chamber programmed for 16/8 h at 25/20°C for day/night, relative humidity of 60–70%. After 30 days, seedlings were transplanted to Jiangpu Horticultural Experimental Station of Nanjing Agricultural University. In March 2015, after removing the already opened flowers and siliques, inflorescences of the main and lateral branch were covered in plastic wrap. The next day, the plants were used for samples collection under normal growth conditions or after pollination treatments. Samples from five plants were treated as a biological replicate and triplicate samples were collected in each analysis. All samples were snap frozen in liquid nitrogen and stored at −80°C until further analysis.

#### Floral development stages (FD)

The criterion of floral development stage can be defined according to the number of days until flower opening (Stein et al., 1996). We describe them as follows. Floral development stage 1 (FD1): Flower buds at 5 days before anthesis. The lengths of the flower buds are approximately 3 mm. Floral development stage 2 (FD2): Flower buds at 3 days before anthesis with lengths of approximately 5 mm. Floral development stage 3 (FD3): Flower buds at 1 day before anthesis. The lengths of the flower buds are 7 mm. Floral development stage 4 (FD4): Freshly opening flowers. Flowers from SI and SC plant were defined as FD_*I*_1-4 and FD_*C*_1-4, respectively.

#### Stigma development stages (SD)

Stigmas were randomly collected from approximately 150 flower buds or freshly opened flowers corresponding to the four stages mentioned above. Stigmas from SI and SC plant were defined as SD_*I*_1-4 and SD_*C*_1-4, respectively.

#### Floral tissues (FT)

Flower organs were separated from freshly opening flowers with forceps. Five different floral tissues including petals (FT1), sepals (FT2), anther (FT3), stigmas (FT4), and ovary (FT5) were collected. Floral tissues from SI and SC plant were defined as FT_*I*_1-5 and FT_*C*_1-5, respectively.

#### Pollinated stigmas (PS)

Stigmas of freshly opening flowers were pollinated at anthesis with self-pollen and harvested immediately, which was defined as PS1. The other self-pollinated pistils were wrapped for 0.25 and 0.5 h and then were collected as PS2 and PS3. Stigmas from SI and SC plant were defined as PS_*I*_1-3 and PS_*C*_1-3, respectively. The fresh weight of pistils of each group (PS_*I*_1-3and PS_*C*_1-3) amounted to about 200 mg

### Total RNA isolation and cDNA synthesis

Total RNA was extracted by RNA simple Total kit (TaKaRa, Dalian, China), according to the manufacturer's instructions. Then, the RNA was treated with DNase I digestion (TaKaRa, Dalian, China) to eliminate potential DNA contamination. The RNA concentration and purity were determined by Nanodrop ND 1000 spectrophotometer (Nanodrop Technologies, USA). Only RNA samples with A_260_/A_280_ ratio between 1.9 and 2.1 and A_260_/A_230_ ratio higher than 2.0 were used in the analysis. RNA integrity was further assessed by 1.5% agarose gel electrophoresis. Two microgram of total RNA was reverse transcribed into cDNA with a final volume of 20 μL using a Prime Script RT reagent kit (TaKaRa, Dalian, China), according to the manufacturer's instructions.

### Selection of reference genes and primer design

Thirteen candidate reference genes, including eight protein-coding traditional reference genes (*ACT7, EF1*α, *TUB4, GAPDH, CYP, DNAJ, HIS*, and *TUA5*) and five novel reference genes (*ACP, UKN1, SKIP16, CAC*, and *PP2A*), were selected and evaluated for this study from TAIR database (http://www.arabidopsis.org) and previous study (Czechowski et al., 2005). Potential homologs of the 13 reference genes were identified from the genome sequencing data of non-heading Chinese cabbage (unpublished data). These candidate reference genes were cloned and confirmed through sequencing. The primers were designed using Beacon Designer v 7.9 (Premier Biosoft International, Palo Alto, CA, USA) with melting temperatures (TM) of 58–62°C, primer lengths of 18–25 bp and amplicon lengths of 100–250 bp (Table 1).

**Table 1:** **Descriptions of candidate reference genes in *B. rapa***.

**Gene name**	**Accession number**	**Arabidopsis homolog locus**	**Primer sequence (5′−3′) forward/reverse**	**Amplicon length (bp)**	**Primers Tm (°C)**	**E (%)**	**R^2^**
Actin7 (*ACT7*)	KU851921	AT5G09810	CTACGAGTTACCTGATGGA/ATGATGGAGTTGTAAGTTGTC	132	59.4/59.7	101.8	0.998
Elongation factor -1α (*EF1α*)	KU851922	AT1G07940	ATGGTGATGCTGGTATGG/TCCTTCTTGTCAACACTCTT	148	60.6/60.6	101.8	0.998
Beta-tubulin-4 (*TUB4*)	KU851924	AT5G44340	AACAGTACAGTGCCTTGA/GACCTCCTTAGTGCTCAG	143	59.9/60.2	102.4	0.997
Glyceraldehyde 3- phosphate dehydrogenase (*GAPDH*)	KU851923	AT3G04120	GCTGCTTCATTCAACATC/CATCATCCTCGGTGTATC	241	58.2/58.0	105.2	0.995
Cyclophilin (*CYP*)	KU851925	AT2G21130	GAGTCCATCTACGGTGAG/AGCCAATCGGTCTTAACA	140	60.0/60.0	96.9	0.990
DNAJ-like (*DNAJ*)	KU851926	AT3G44110	CATGGAGATAGACGAGTG/CCTTCTTCATCATCATCATC	117	58.1/58.0	100.7	0.986
Histone H3 (*HIS*)	KU851927	AT5G10980	GAAGAAGCCTCACCGTTA/CGAACAGACCCACAAGATA	209	60.4/60.6	110.1	0.997
Alpha tubulin 5 (*TUA5*)	KU851928	AT5G19780	GAGTTCCAGACCAACCTT/CACAGCAGCATTAACATCTT	237	60.3/60.5	95.4	0.998
Acyl carrier proteins 1 (*ACP*)	KU851929	AT3G05020	CAACAACAACGAGGATAAGT/AAGAGATTGAGAGGCGAAT	102	59.8/60.0	102.7	0.992
Hypothetical (*UKN1*)	KU851930	AT3G13410	TAATAGCACCGTTGGAGTT/CACTGATGAGGATGAGAAGA	110	60.3/60.3	105.6	0.992
SKP1/Ask-interacting protein 16 (*SKIP16*)	KU851931	AT1G06110	CTCAACATCACTACTCCTCTC/AATGGCTAACACGCTTCA	124	61.1/61.1	97.5	0.996
Clathrin adaptor complex (*CAC*)	KU851932	AT5G46630	CTGCTCCTTCGTCTACAT/AGTCCATAATCTCGTCTAACA	200	59.9/59.8	97.3	0.996
Protein phosphatase 2A (*PP2A*)	KU851933	AT1G10430	ACCGTGGCTACTATTCAG/GCAGTAAGAGGAAGATAATCG	208	59.9/59.7	104.1	0.994

### Quantitative RT-qPCR assay

RT-qPCR was performed in 96-well optical plates (Bio-Rad, USA) on the StepOnePlus™ System (Applied Biosystems®, Foster City, CA, USA). The reaction mixture contained 10 μL of 2 × SYBR® Premix Ex Taq™ II (TaKaRa, Dalian, China), 0.8 μL of each primer (10 μM), 0.4 μL of 50 × ROX Reference Dye, 2 μL of diluted cDNA (1:10), and 6 μL of dH_2_O in a final volume of 20 μL. Amplifications were performed with the following cycling profile: 95°C for 30 s, 40 cycles of 95°C for 5 s and 60°C for 30 s, and a melting curve analysis protocol (60–95°C with temperature increment of 0.2°C every 10 s). No-template controls were also done at the same time.

All RT-qPCR experiments were performed with three biological and three technical replicates. Amplification efficiency (E) and correlation coefficient (R^2^) were calculated using the SDS software version 2.3 (Applied Biosystems®, Foster City, CA, USA) by standard curve method with tenfold serial dilutions method.

### Data analysis

The expression stability of the 13 reference genes were evaluated by geNorm and NormFinder in 96 samples (three biological replicates and 32 different conditions) under different experimental conditions. Expression levels of the tested reference genes were represented by Cq values, the cycle number at which the amplification curve reach a specific threshold. The raw Cq data (Table S1) were firstly transformed into relative expression levels with efficiency of each primer pairs according to previous research (Ramakers et al., 2003; Xiao et al., 2014). The transformed data were then used for the geNorm and the NormFinder, respectively. Moreover, sometimes reference genes may have different ranking in geNorm and NormFinder, to obtain the most stable reference genes, geometric mean was used to obtain a final result (Štajner et al., 2013; Xiao et al., 2014).

The geNorm is a Visual Basic application software and relies on the principle that the logarithmically transformed expression ratio of two perfect reference genes should be constant in a given sample set. The candidate reference genes were ranked according to the expression stability value M, which is the average pair-wise variation of a particular gene with all other reference genes. The gene with the lower M value is considered to have more stable expression, while that with the higher M value has the less stable expression (Vandesompele et al., 2002). Additionally, the geNorm can also calculates (V_n∕n+1_), which is the pairwise variation of two sequential normalization factors NF_n_ and NF_n+1_, to determine the minimum number of reference genes for accurate normalization with a cut-off value of 0.15 (Vandesompele et al., 2002).

NormFinder program is another Visual Basic application software used to assess the expression stabilities of tested genes according to intra- and inter-group variations and assigned a value for each candidate reference gene as a measure of their stability. The genes with lower values indicate low intra- and inter-group variations and are consider to have greater stability (Andersen et al., 2004).

### Validation of identified reference gene

To further validate the present analysis, the relative expression profiles of *SRK* (in different stigma development stages and different floral tissues of SI line) and *Exo70A1* (in different floral tissues of SI and SC lines) were normalized by the most/least stable reference genes according to geNorm and NormFinder analysis. The RT-qPCR experimental procedure were the same as those described above. Data were calculated and analyzed using SDS version 2.3 software with the 2^−ΔΔCt^ method (Livak and Schmittgen, 2001). The raw Cq values were listed in Table S2.

## Results

### Assessment of efficiency and specificity of reference genes

PCR amplification specificity for each gene was evaluated by agarose gel electrophoresis, with cDNA and genomic DNA as templates (Figure S1). The results demonstrated that all thirteen primer pairs amplified a specific product with the expected size and no primer dimers were found. The amplification of *UKN1* and *SKIP16* differed in size between genomic DNA and cDNA templates, as there are introns between the primer pair sequences on the genome DNA (Figure S1). These two genes were then used for assessing RNA quality. Specific amplifications were also confirmed by the melting curve analyses which showed a single and dominant peak for all the primer pairs (Figure S2).

All RNA samples were verified by PCR results of *UKN1* and *SKIP16* before carrying out RT-qPCR experiments. There was only a specific product with the expected size derived from cDNA template for each sample, which indicated there was no genomic DNA contamination in cDNA templates (data not shown). The RT-qPCR efficiency of the 13 reference genes varied from 95.4% for *TUA5* to 110.1% for *HIS*, and correlation coefficients ranged from 0.986 to 0.998 (Table 1).

### Expression profile of the reference genes

**Figure 1:**
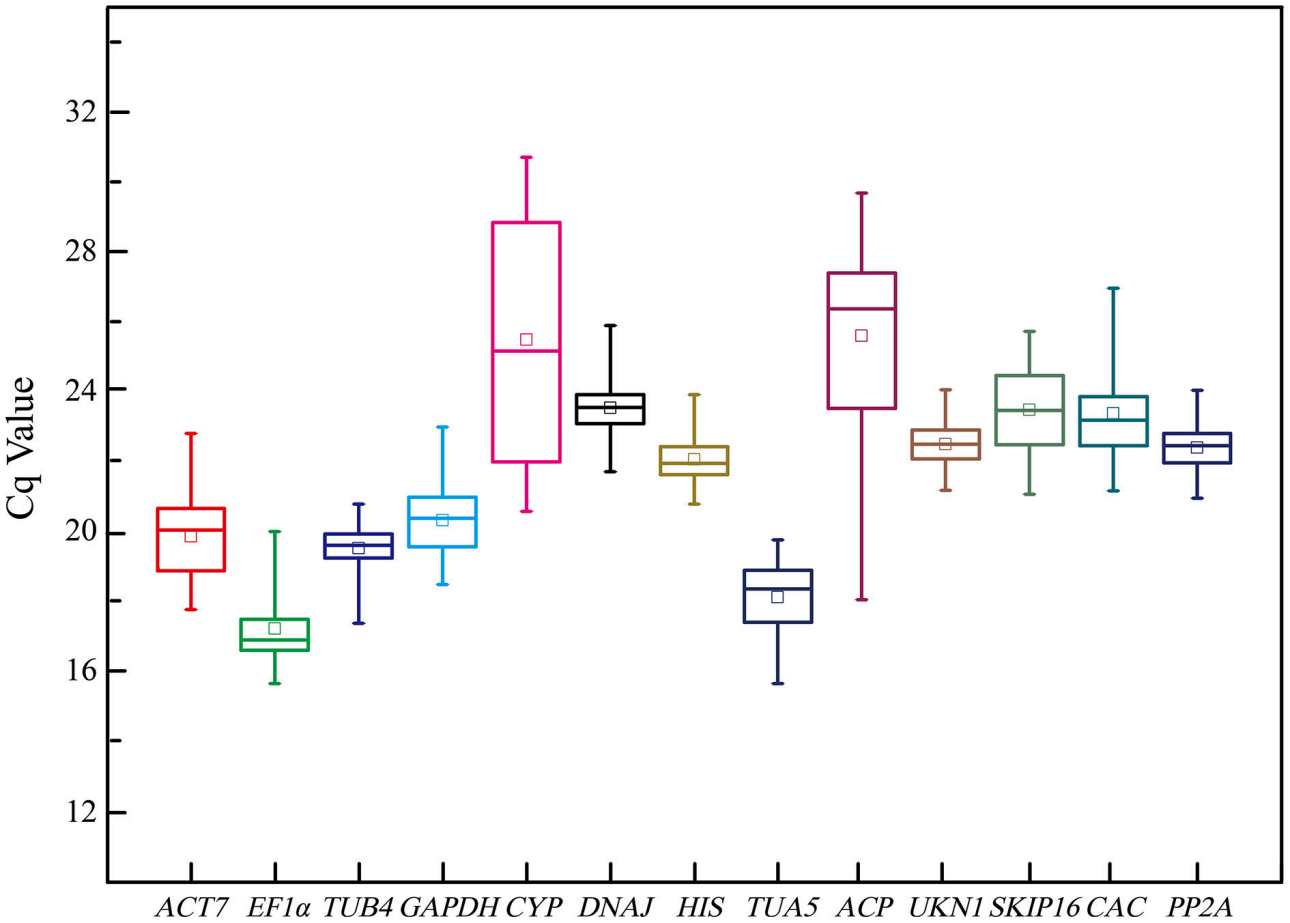
**Cq values of 13 candidate reference genes tested in all 96 samples of non-heading Chinese cabbage**. The line across the box depicts median. The inside box depicts mean. The outside box is determined by the 25th and 75th percentiles. The whiskers indicate the maximum and minimum values.

**Figure 2:**
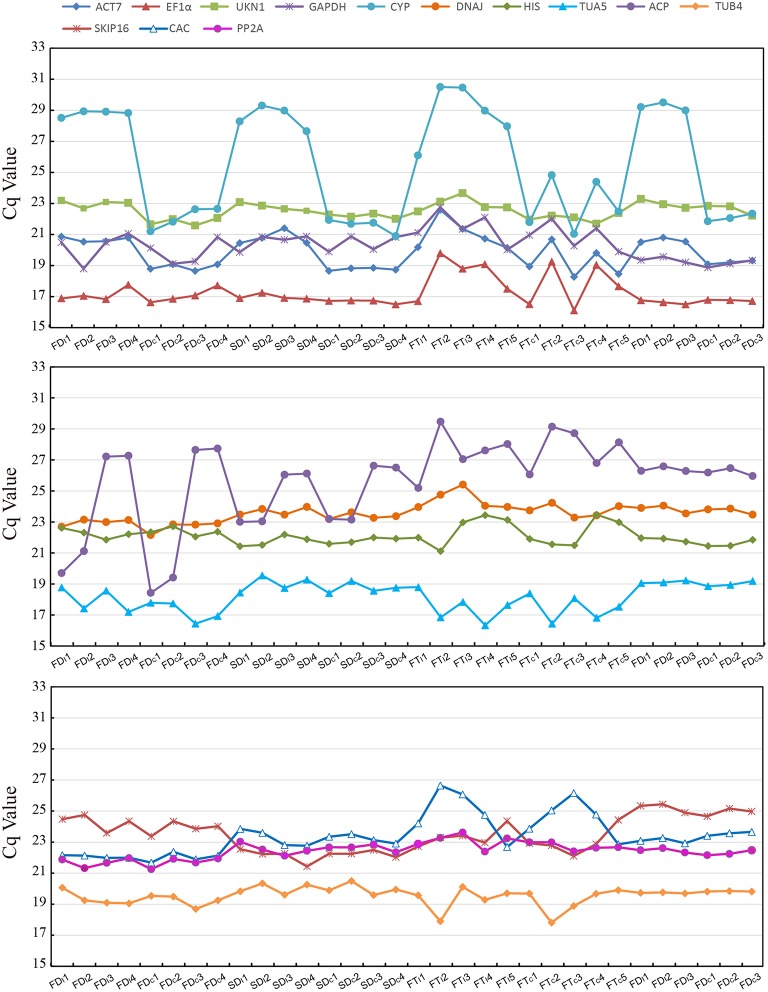
**The mean Cq values of 13 candidate reference tested in all 32 conditions**.

Figure 1 provide an overview of the transcript levels of all 13 candidate reference genes. The expression levels of the candidate reference genes were determined as quantification cycle (Cq) values, and the transcripts abundance of these genes varied widely. Mean Cq values of all 32 conditions ranged from 17.38 *(EF1*α*)* to 25.62 (*CYP*) across all samples, while the majority of Cq values were between 17 and 25 (Figure 1). Interestingly, our data showed that *CYP* had a relatively low expression (high Cq values) in all SI samples than SC samples and *ACP* had a low expression 0 and 1 day before flowering than 3 and 5 days before flowering in both flowers (flower buds) and stigmas (Figure 2), so these two genes showed the most variation in expression levels (Figure 2). In addition, *CYP* and *ACP* showed relatively low expression levels with high Cq values, whereas, *ACT7, EF1*α*, TUB4, GAPDH*, and *TUA5* showed high expression. Most candidate genes have close mean values and median values, especially for *DNAJ, UKN1*, and *SKIP16*, indicating evenly distributed Cq values (Figure 2).

### Expression stability of candidate reference genes in two *line*s

After simple comparison of the Cq values of the potential reference genes, the stability of these genes was further assessed using geNorm and NormFinder. All 96 samples were divided into five groups: different flower developmental stages, different stigma developmental stages, various floral tissues, stigmas after self-pollination and total (data from all 96 samples), and the condition-specific reference genes were identified for each group.

As shown in Table 2, all reference genes were ranked based on their expression stability. For all 96 samples tested, *DNAJ, UKN1*, and *PP2A* were the three most stabe reference genes in both geNorm and NormFinder analysis. In different flower developmental stages, the most stable reference genes identified by geNorm differ from that defined by NormFinder, *DNAJ* was the most stable by a comprehensive comparison. In different stigma developmental stages, *EF1*α*, DNAJ, and TUA5* were ranks as the top three reference genes among the 13 candidate genes. *DNAJ* and *UKN1* were the two most stable genes among the 13 candidate genes in different floral tissues. In stigmas after self-pollination, *GAPDH* and *HIS* were the two most stable genes even though other 11 candidate genes also expressed stably. *CYP* was proved to be the least stable genes in all five condition-specific groups, as this genes had a relatively low expression in SI samples (average Ct value was 28.8) than SC samples (average Ct value was 22.2).

**Table 2:** **Gene expression stability under individual sample sets in two *lines* calculated by geNorm and NormFinder**.

**Gene**	**geNorm**					**NormFinder**				
	**Floral development stages**	**Stigma development stages**	**Floral tissues**	**Pollinated Stigmas**	**Total**	**Floral development stages**	**Stigma development stages**	**Floral tissues**	**Pollinated Stigmas**	**Total**
*ACT7*	0.61 (9)	0.53 (10)	1.03 (8)	0.37 (11)	0.90 (6)	0.30 (5)	0.34 (8)	0.53 (5)	0.18 (8)	0.40 (4)
*EF1α*	0.40 (5)	0.29 (3)	1.09 (9)	0.24 (5)	0.84 (5)	0.24 (3)	0.09 (2)	0.62 (7)	0.28 (11)	0.40 (5)
*TUB4*	0.43 (6)	0.25 (2)	0.72 (4)	0.22 (4)	0.69 (3)	0.49 (10)	0.30 (6)	0.78 (9)	0.26 (10)	0.65 (8)
*GAPDH*	0.66 (10)	0.45 (9)	0.95 (7)	0.19 (2)	0.94 (7)	0.45 (8)	0.30 (7)	0.50 (4)	0.05 (1)	0.56 (6)
*CYP*	1.08 (11)	1.15 (12)	1.50 (12)	0.84 (12)	1.70 (12)	2.13 (12)	2.33 (13)	2.03 (13)	2.37 (13)	2.25 (13)
*DNAJ*	0.31 (2)	0.24 (1)	0.34 (1)	0.26 (7)	0.41 (1)	0.08 (1)	0.08 (1)	0.08 (1)	0.18 (9)	0.09 (1)
*HIS*	0.37 (4)	0.43 (8)	0.86 (6)	0.17 (1)	0.76 (4)	0.49 (9)	0.27 (5)	0.74 (8)	0.05 (2)	0.56 (7)
*TUA5*	0.55 (8)	0.24 (1)	0.79 (5)	0.20 (3)	1.05 (9)	0.56 (11)	0.20 (4)	0.85 (11)	0.17 (7)	0.80 (10)
*ACP*	1.55 (12)	0.74 (11)	1.22 (11)	0.26 (8)	1.41 (11)	2.80 (13)	1.26 (12)	0.95 (12)	0.16 (5)	1.96 (12)
*UKN1*	0.49 (7)	0.34 (4)	0.34 (1)	0.30 (10)	0.56 (2)	0.12 (2)	0.09 (3)	0.11 (2)	0.17 (6)	0.11 (2)
*SKIP16*	0.35 (3)	0.41 (7)	0.57 (3)	0.25 (6)	1.12 (10)	0.36 (7)	0.37 (11)	0.55 (6)	0.06 (3)	0.89 (11)
*CAC*	0.29 (1)	0.39 (6)	1.15 (10)	0.28 (9)	1.00 (8)	0.33 (6)	0.36 (10)	0.82 (10)	0.41 (12)	0.73 (9)
*PP2A*	0.29 (1)	0.38 (5)	0.41 (2)	0.17 (1)	0.41 (1)	0.26 (4)	0.35 (9)	0.28 (3)	0.10 (4)	0.32 (3)

### Expression stability of candidate reference genes in single *line*

Some studies may only evaluate the expression of reference genes in a single *line*, meanwhile, to investigate the effect of different *line*s to the ranks of reference genes, we decided to perform stability analysis using the geNorm and NormFinder software programs using only the SI line or SC line as input. Accordingly, a total of 10 evaluation patterns were generated for both single experimental condition and total (all 48 SI samples or all 48 SC samples). As a result, *DNAJ, UKN1*, and *PP2A* were also the three most stable genes in all SI samples or all SC samples for both geNorm and NormFinder analysis, which was the same as above analysis in all 96 samples (Table 3). For stigmas after self-pollination in SI line or SC line, all reference genes had low M values in geNorm analysis and low stability values in NormFinder analysis, which indicated they were all expressed stably in both groups. For the other six groups in single inbred line, the best ranks of the reference genes were changed considerably comparing with the above analysis in two inbred lines. In SI plant, the two most stable reference genes for all of the experimental conditions determined were almost the same between geNorm and NormFinder analysis. In different flower developmental stages, *ACT7* and *PP2A* were the two most stable reference genes. In different stigma developmental stages, *ACT7* and *EF1*α were the two most stable reference genes. *DNAJ* and *UKN1* were the two most stable reference genes in different floral tissues. In SC plant, *DNAJ* and *PP2A* performed well in different floral tissues by analysis with both software. For the other two groups, it was difficult to identify the most stable reference gene, so the geometric mean was used to obtain a final result (Štajner et al., 2013; Xiao et al., 2014). Finally, *UKN1* and *PP2A* were selected as the two most stable reference genes in different flower developmental stages. In stigma at different developmental stages, *ACT7* and *SKIP16* were selected as the most stable genes.

**Table 3:** **Gene expression stability under individual sample sets in single *line* calculated by geNorm and NormFinder**.

**Species**	**Gene**	**geNorm**					**NormFinder**				
		**Floral development stages**	**Stigma development stages**	**Floral tissues**	**Pollinated Stigmas**	**All**	**Floral development stages**	**Stigma development stages**	**Floral tissues**	**Pollinated Stigmas**	**All**
SI	*ACT7*	0.22 (1)	0.13 (1)	0.59 (3)	0.13 (1)	0.59 (3)	0.07 (1)	0.05 (2)	0.39 (4)	0.08 (4)	0.24 (2)
	*EF1α*	0.36 (7)	0.13 (1)	0.73 (4)	0.19 (10)	0.81 (6)	0.15 (6)	0.02 (1)	0.53 (6)	0.11 (9)	0.46 (5)
	*TUB4*	0.41 (9)	0.23 (3)	0.94 (7)	0.17 (7)	0.70 (4)	0.43 (9)	0.19 (4)	0.77 (8)	0.09 (5)	0.62 (8)
	*GAPDH*	0.55 (11)	0.44 (9)	0.79 (5)	0.17 (6)	0.91 (8)	0.44 (11)	0.23 (7)	0.58 (7)	0.10 (7)	0.62 (9)
	*CYP*	0.29 (3)	0.49 (11)	1.17 (11)	0.15 (3)	0.86 (7)	0.11 (5)	0.43 (12)	0.85 (11)	0.10 (8)	0.57 (7)
	*DNAJ*	0.30 (5)	0.22 (2)	0.32 (1)	0.16 (4)	0.40 (1)	0.10 (4)	0.10 (3)	0.16 (2)	0.10 (6)	0.16 (1)
	*HIS*	0.33 (6)	0.46 (10)	1.05 (9)	0.16 (5)	0.76 (5)	0.36 (8)	0.20 (6)	0.83 (10)	0.07 (2)	0.54 (6)
	*TUA5*	0.48 (10)	0.26 (4)	1.00 (8)	0.20 (11)	1.03 (10)	0.53 (12)	0.24 (8)	0.87 (13)	0.15 (12)	0.86 (11)
	*ACP*	1.02 (12)	0.68 (12)	1.22 (12)	0.18 (8)	1.33 (12)	2.48 (13)	1.17 (13)	0.86 (12)	0.11 (10)	1.69 (13)
	*UKN1*	0.29 (4)	0.31 (5)	0.32 (1)	0.22 (12)	0.56 (2)	0.08 (3)	0.20 (5)	0.10 (1)	0.18 (13)	0.25 (3)
	*SKIP16*	0.39 (8)	0.41 (8)	0.85 (6)	0.18 (9)	1.11 (11)	0.44 (10)	0.34 (10)	0.51 (5)	0.12 (11)	0.91 (12)
	*CAC*	0.24 (2)	0.38 (7)	1.11 (10)	0.14 (2)	0.96 (9)	0.18 (7)	0.39 (11)	0.76 (9)	0.08 (3)	0.71 (10)
	*PP2A*	0.22 (1)	0.35 (6)	0.39 (2)	0.13 (1)	0.40 (1)	0.07 (2)	0.31 (9)	0.31 (3)	0.06 (1)	0.26 (4)
SC	*ACT7*	0.12 (1)	0.16 (4)	0.93 (8)	0.12 (1)	0.54 (3)	0.17 (7)	0.06 (1)	0.47 (5)	0.06 (1)	0.29 (4)
	*EF1α*	0.30 (7)	0.14 (3)	1.00 (9)	0.18 (7)	0.66 (4)	0.12 (3)	0.08 (3)	0.73 (9)	0.07 (4)	0.46 (5)
	*TUB4*	0.33 (8)	0.29 (9)	0.67 (4)	0.17 (6)	0.85 (8)	0.44 (10)	0.27 (10)	0.71 (8)	0.07 (3)	0.60 (9)
	*GAPDH*	0.49 (11)	0.35 (11)	0.88 (7)	0.14 (4)	0.81 (7)	0.44 (11)	0.33 (12)	0.43 (4)	0.14 (8)	0.59 (8)
	*CYP*	0.38 (9)	0.31 (10)	1.07 (10)	0.15 (5)	0.71 (5)	0.13 (5)	0.32 (11)	0.85 (11)	0.14 (9)	0.55 (7)
	*DNAJ*	0.25 (6)	0.22 (6)	0.27 (1)	0.22 (10)	0.36 (1)	0.08 (2)	0.09 (5)	0.17 (2)	0.16 (10)	0.03 (1)
	*HIS*	0.20 (3)	0.24 (7)	0.80 (6)	0.19 (8)	0.76 (6)	0.33 (9)	0.07 (2)	0.60 (7)	0.17 (11)	0.52 (6)
	*TUA5*	0.43 (10)	0.26 (8)	0.74 (5)	0.12 (2)	0.97 (10)	0.64 (12)	0.19 (8)	0.75 (10)	0.10 (5)	0.78 (11)
	*ACP*	1.11 (12)	0.57 (12)	1.14 (11)	0.24 (11)	1.34 (12)	3.12 (13)	1.26 (13)	0.86 (12)	0.18 (12)	2.03 (13)
	*UKN1*	0.12 (1)	0.13 (2)	0.33 (2)	0.26 (12)	0.44 (2)	0.12 (4)	0.10 (6)	0.26 (3)	0.27 (13)	0.28 (3)
	*SKIP16*	0.22 (4)	0.10 (1)	0.52 (3)	0.20 (9)	1.03 (11)	0.17 (6)	0.08 (4)	0.55 (6)	0.13 (7)	0.81 (12)
	*CAC*	0.18 (2)	0.20 (5)	1.19 (12)	0.12 (1)	0.92 (9)	0.17 (8)	0.21 (9)	0.86 (13)	0.07 (2)	0.65 (10)
	*PP2A*	0.24 (5)	0.10 (1)	0.27 (1)	0.13 (3)	0.36 (1)	0.08 (1)	0.11 (7)	0.16 (1)	0.10 (6)	0.17 (2)

### Minimum number of reference genes for optimal normalization

**Figure 3:**
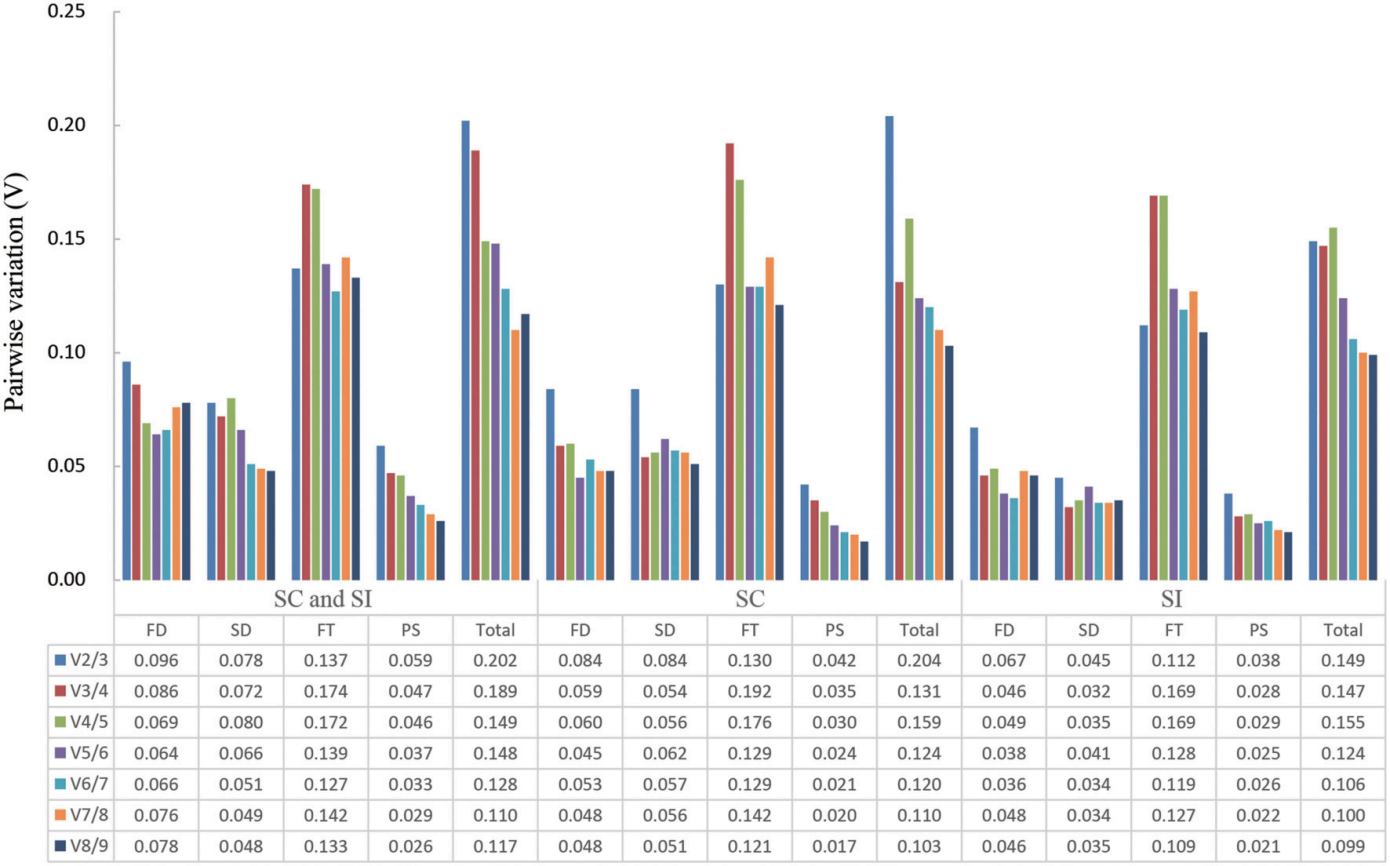
**Determination of the optimal number of reference genes required for effective normalization**. Pairwise variation (Vn/n + 1) analysis of 13 candidate reference genes in 15 sample sets. FD, different floral development stages; SD, different stigma development stages; FT, different floral tissues; PS, pollinated stigmas; Total, all samples.

The pairwise variation V_n_/V_n+1_ (n ≥ 2) of geNorm software, which views the effect of increasing number of genes on the normalization factor (Pfaffl et al., 2004), was used to determine the minimal number of reference genes required for accurate normalization in all 15 sample sets. Results showed that only two sample sets, the 96 samples group and the 48 SC line samples group, had a V_2∕3_ value higher than 0.15 (Figure 3). Thus, four and three reference genes were needed for gene expression normalization in the 96 samples and the 48 SC line samples, respectively. For other 13 conditions, two reference genes are sufficient for normalizing gene expression.

### Reference gene validation

To validate the reference genes confirmed above, the relative expression profiles of *SRK*, which is expressed specifically in stigma (Stein et al., 1996) and *Exo70A1*, which was reported to be without significant difference in expressions level between compatible and incompatible floral tissues (Wang et al., 2014b) were measured and normalized with three sets of reference genes according to the rankings of reference genes mentioned above. Briefly, the most stable reference genes (*ACT7* and *EF1*α for stigma samples of SI at different developmental stages, *DNAJ* and *UKN1 for* different floral tissues of SI samples and SC-SI mixed samples) either alone or combination, and the least stable reference gene (*ACP* for stigma samples of SI at different developmental stages, *CYP* for different floral tissues of SI samples and SC-SI mixed samples), were used as reference genes for RT-qPCR analysis.

**Figure 4:**
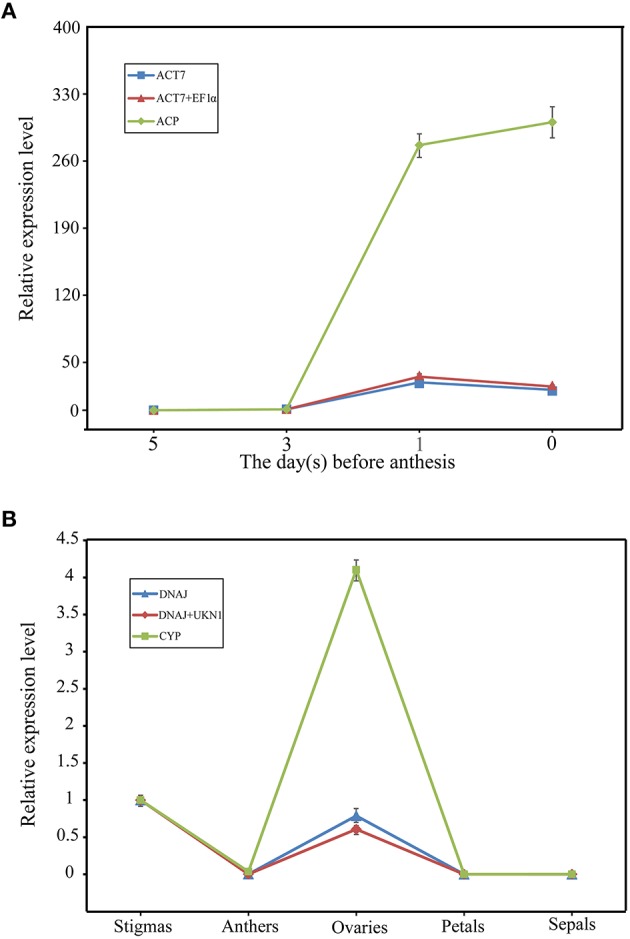
**Relative quantification of *SRK* gene expression using validated reference genes for normalization under different experimental conditions. (A)** The validated reference gene(s) used as normalization factors were one (*ACT7*) or two (*ACT7* + *EF1*α) most stable reference genes, and the most unstable one (*ACP*) in different stigma development stages sample sets of SI plant. **(B)** The validated reference gene(s) used as normalization factors were one (*DNAJ*) or two (*DNAJ* +*UKN1*) most stable reference gene(s), and the most unstable one (*CYP*) in different floral tissues sample sets of SI plant. Vertical bars indicate the standard deviations (SD).

As shown in Figure 4, SRK transcripts increased during stigma maturation and reached a maximum at 1 day before flowering in SI plant, showed similar change patterns with slight difference when using *ACT7* alone and the combination of *ACT7* + *EF1*α as reference gene(s) for normalization (Figure 4A). *SRK* expressed mainly in stigma, rather than in anther, ovary, petal and sepal, with the normalization of *DNAJ* alone and the combination of *DNAJ* + *UKN1* (Figure 4B). However, the expression patterns were completely obscured during normalization using the least stable reference gene *ACP* in stigma or *CYP* in different floral tissues.

**Figure 5:**
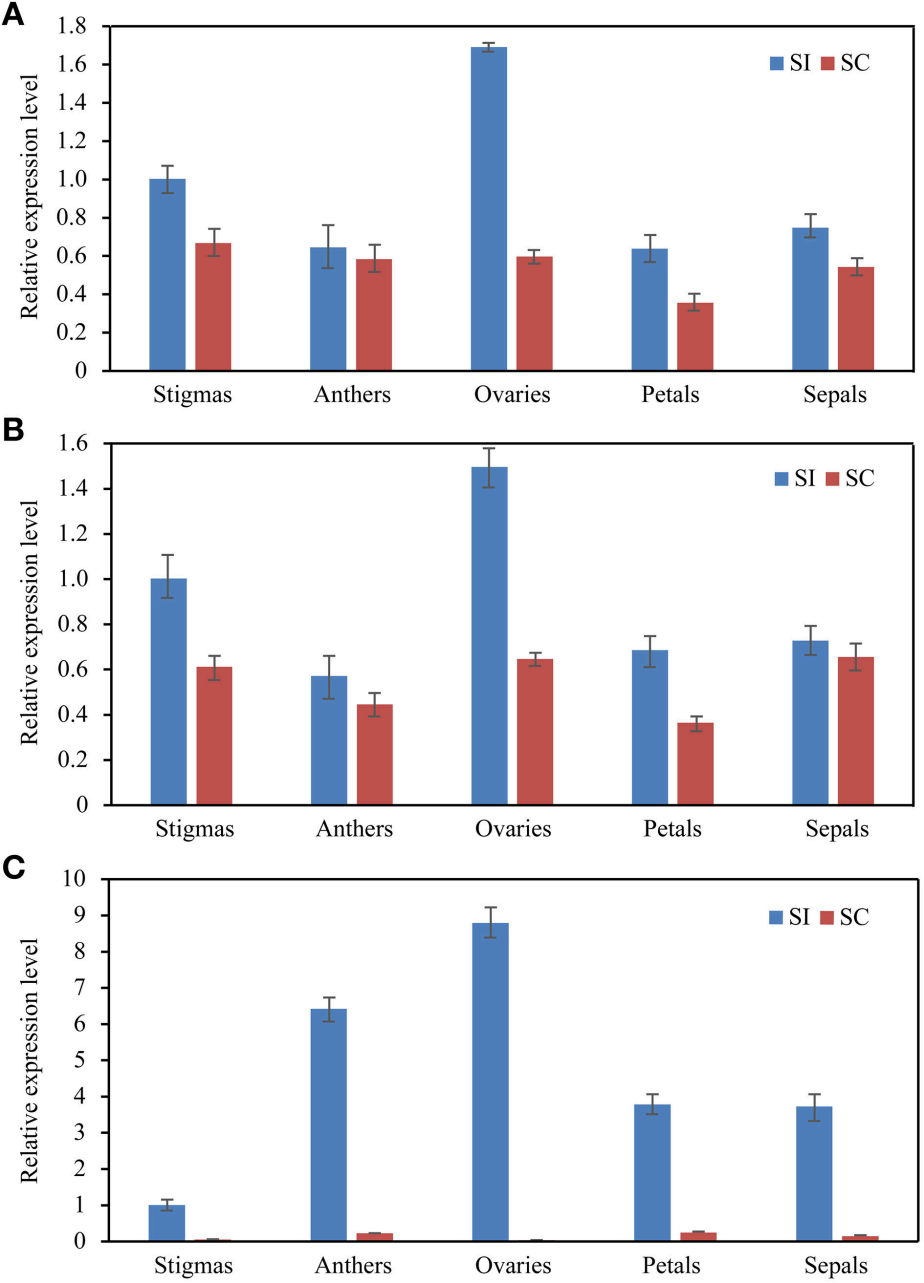
**The expression level of the *Exo70A1* in floral tissue sample sets**. *DNAJ* **(A)** or *DNAJ* + *UKN1* **(B)** were used for normalization as one or two most stable reference genes, *CYP* **(C)** was used as worst stable reference gene.

To further explore the stability of these reference genes, the expression levels of *Exo70A1* was also investigated in different floral tissues of SI and SC lines. *Exo70A1* is a component of the exocyst complex that is known to regulate polarized secretion (Samuel et al., 2009). Mutations of *Exo70A1* led to aberrant development, producing dwarfed and nearly sterile (Li et al., 2013). As shown in Figure 5, when using *DNAJ* alone and the combination of *DNAJ* + *UKN1*for normalization, *Exo70A1* expressed in all floral tissues of SI and SC lines(Figures 5A,B); however, when the least stable gene *CYP* was used for normalization, the expression patterns was absolutely different. *Exo70A1* had high expression level in SI line and almost had no expression in SC line (Figure 5C).

## Discussion

Non-heading Chinese cabbage is an ideal model plant for self-incompatibility research and is also an important Brassica crop. Analysis of the expression of genes involved in the signaling systems regulating compatible and self-incompatible responses would aid our understanding of the molecular mechanism of SI in Brassica. Due to the high throughput, sensitivity and accuracy, RT-qPCR has become a powerful approach for quantification studies at transcript levels (Artico et al., 2010). The expression stability of reference genes is an elementary prerequisite for reliable RT-qPCR analysis. However, no single reference gene can be universal under all experimental situations, even the most stable reference gene(s) reported in a species may react badly in other species or in a different experiment (Kim et al., 2003; Argyropoulos et al., 2006; Fu et al., 2013; Niu et al., 2015). Therefore, it is required to perform normalization studies with multiple candidate genes under particular experimental conditions prior to applying them to normalization, instead of using reference genes published previously. In this study, we performed a systematic evaluation of 13 reference genes in four strategic groups in compatible and self-incompatible lines of non-heading Chinese cabbage, and ranked them according to their stability calculated by geNorm and NormFinder. The present study was the first attempt to validate the reference genes direct at SI in Brassica.

Previous study has suggested that amplification-specificity of primers should be validated with direct experimental evidence (agarose gel electrophoresis, melting profile, sequencing etc.; Bustin et al., 2009; Chen et al., 2011). In the present work, all 13 genes were cloned from non-heading Chinese cabbage and confirmed through sequencing. Primer pairs were designed for the 13 candidate reference genes, and their specificity was confirmed by agarose gel electrophoresis (Figure S1), melting curves analysis (Figure S2), and sequencing of the amplicons. Subsequently, the PCR amplification efficiency was calculated according to corresponding standard curves, which ranged from 95.4 to 110.1%. Other factors that may affect the reliability of the data were also carefully controlled during our experiments, such as DNAse I treatment, RNA quality control, biological replicates and correction raw results. Our results confirmed that all materials used in this study were appropriate for qPCR-based quantification.

Our analysis based on geNorm and NormFinder algorithms showed *DNAJ, UKN1*, and *PP2A* were overall the most stable candidates for the normalization of target gene expression across total 96 samples. But different sample sets had their own most stable reference genes (Tables 2, 3). As show in the Table 2, *EF1*α*, DNAJ, and TUA5* were the most stable genes in stigma at different development stages, whereas *GAPDH* and *HIS* ranked higher than *EF1*α*, DNAJ, and TUA5* when studying stigmas samples after self-pollination. *DNAJ, UKN1*, and *PP2A* appeared to be the most stable reference genes in different floral tissues samples, while *UKN1* and *PP2A* ranked lower in stigma samples at different developmental stages. For the most stable reference genes in single inbred line, all 10 samples sets had their own most stable reference genes (Table 3). As a whole, our results indicated that different experimental condition tested demands different reference genes, emphasized the importance of reference genes identification before RT-qPCR analysis. Earlier studies also reported similar results in other species, including *peanut* (Chi et al., 2012)*, lettuce* (Borowski et al., 2014), *and watermelon* (Kong et al., 2014). More importantly, our result showed that the choice of reference genes for normalization should be line-specific. Even the samples belong to same type and is from same species (but belong to different lines), they may have different set of reference genes when they were analyzed alone or together. For instance, for samples from stigma at different developmental stages, *ACT7* and *EF1*α were the most stable reference genes in SI plant, *SKIP16* was the most stable gene in SC plant, whereas *EF1*α*, DNAJ, and TUA5 were the* most stable reference genes when analyzed together. This result suggested that different experimental condition tested demands different set of reference genes, even when samples come from very closely related *lines*.

In the study of different flower developmental stages, *ACT7* and *PP2A* were the two best reference genes in SI plant, while *UKN1* and *PP2A* were selected as the two best reference genes in SC plant. Similar studies were also conducted in other species, such as Chinese cabbage (Xu et al., 2014), *Petunia hybrid* (Mallona et al., 2010), *Chrysanthemum lavandulifolium* (Fu et al., 2013) and a combination analysis of *Chrysanthemum lavandulifolium* and *Chrysanthemum morifolium* (Qi et al., 2016). In Chinese cabbage, one of the most stable reference gene was determined as *GAPDH* (Xu et al., 2014), which was one of the least stable reference genes in both SI and SC line in our study. In *P. hybrid* (Mallona et al., 2010), *EF1a* and *CYP* were determined as the most stable reference genes in two different lines, respectively. While this two genes didn't perform well in both SI and SC lines in our study. *SKIP16* were selected as one of the most stable reference gene in *C. lavandulifolium* (Fu et al., 2013), but this gene ranked very low in our study. For the combination analysis of *C. lavandulifolium* and *C. morifolium, PP2A* was determined as the least stable reference gene in most of their condition (Qi et al., 2016), but this gene was the most stable gene in both SC and SI lines in our study. These results highlight the fact that there is not a “common” reference gene which could be used in all similar studies. Reference gene should selected according to different experimental condition, rather than using them published elsewhere.

*ACP* and *CYP* are two commonly accepted reference genes for gene expression analysis in many plant species (Mallona et al., 2010; Chandna et al., 2012), due to the fact that they were used for many years in RT-qPCR assays. However, recently study have demonstrated that these two gene are not always stable under different experimental conditions (Chen et al., 2011; Chandna et al., 2012; Chi et al., 2012; Wei et al., 2013). For example, Wei et al. demonstrated that *CYP* appeared to be the worst gene to use as reference gene during the ABA treatments. This could be explained partly by the fact that reference genes are implicated in multiple cell metabolism. In this study, for all combined analysis of SI and SC sample sets, *CYP* ranked as the least stable gene for all sample set, which due to it had a significantly expressive discrepancy between SI samples and SC samples (Figure 2). Our validation experiment showed that the use of *CYP* as reference gene in different floral tissues of SI and SC plant leaded to misinterpretation of the *Exo70A1* expression level (Figures 5A–C). For *ACP*, it was found as the most unstable reference genes when was compared in flower and stigma at different developmental stages in SI plant, as *ACP* had a lower expression at 0 and 1 days before anthesis than 3 and 5 days before anthesis in both flowers (flower buds) and stigmas (Figure 2). The using of *ACP* for normalization also led to misinterpretation of the *SRK* expression level. Based on above information, we will not suggest these two genes. Especially, we should pay special attention to genes like *CYP*, which showed significant differential expression between SI and SC plant. The use of such reference genes in closely related lines without prior verification of their expression stability would lead to inaccurate data interpretation and thus generate incorrect results (Figures 5A–C). On the other hand, our results further confirmed the importance of reference genes evaluation prior to experimental applications, and no single reference gene was observed with constant expression in all the tested conditions.

Some studies that exploited both geNorm and NormFinder have showed minor differences in gene ranking and in the evaluation of the most stable ones (Cruz et al., 2009; Boava et al., 2010; Lee et al., 2010), while others have reported distinct discrepancies (Paolacci et al., 2009; Li et al., 2011). In the present work, although the ranking orders of gene stability generated by geNorm and NormFinder were not exactly the same, but the least stable genes recommended by the two methods were the same in all the samples set. In addition, only few differences were observed if the gene sets only considered the most stable ones. For instance, *ACT7, PP2A, UKN1*, and *DNAJ* were the most stably expressed genes in different floral development stages in SI plant when calculated using NormFinder, whereas in geNorm analysis, also *ACT7, PP2A*, and *UKN1* were identified as the three most stable genes, but *DNAJ* was been gave different output for ranking five. The discrepancies between these two methods were expected because they are established on distinct statistical approach. geNorm selects two genes displaying a low intra-group variation and approximately the same non-vanishing inter-group variation (Pfaffl et al., 2004), whereas NormFinder selects the two most stable genes through using intra- and inter-group variations for normalization factor calculations (Andersen et al., 2004).

In summary, the current study evaluated the expression stability of 13 candidate genes across 96 given samples through two commonly used applications. Our results suggested that appropriate reference genes for normalization should be identified according to special experimental conditions. The expression analysis of *SRK* and *Exo70A1* emphasized the importance of selecting proper reference genes for RT-qPCR analysis. Our study is the first attempt to validate the reference genes direct at self-incompatibility in Brassica for the normalization of gene expression analysis. Nevertheless, it provides a preliminary exploration on validation of reference genes for SI study and lays the foundation for the similar study in other species.

## Author contributions

CW, YL conceived and designed the experiments; CW, HC, and TH performed the experiments and collected the data; YL, XH obtained funding and XH contributed reagents/materials/analysis tools; CW, TL analyzed the data; CW wrote the paper; CW, YL revised the paper.

### Conflict of interest statement

The authors declare that the research was conducted in the absence of any commercial or financial relationships that could be construed as a potential conflict of interest.

## Supplementary material

Figure S1 **Amplification results of candidate genes with cDNA and genomic DNA of non-heading Chinese cabbage, respectively**. Lane 1 *ACT7*, 2 *EF1*α, 3*TUB4*, 4 *GAPDH*, 5 *CYP*, 6 *DNAJ*, 7 *HIS*, 8 *TUA5*, 9 *ACP*, 10 *UKN1*, 11 *SKIP16*, 12 *CAC*, 13 *PP2A*. **(A)** The amplification results of 13 candidate genes with cDNA as templates. **(B)** The amplification results of 13 candidate genes with genomic DNA as templates. M: DL 2000 Plus DNA Marker (from up to low, 2000 bp, 1500 bp, 1000 bp, 750 bp, 500 bp, 250 bp, 100 bp).

Figure S2 **Melting curve analysis of 13 reference genes with the single peak**.

Table S1 **Raw Cq values in non-heading Chinese cabbage**. FD, different floral development stages; SD, different stigma development stages; FT, different floral tissues; PS, pollinated stigmas.

Table S2 **Raw Cq values of *SRK, Exo70A1* and reference genes under different conditions, as determined by RT-qPCR**. SD, different stigma development stages; FT, different floral tissues.
