# Supplementary figures and images for: Identification and Validation of Reference Genes for RT-qPCR Analysis in Non-Heading Chinese Cabbage Flowers
Source: Front Plant Sci. 2016 Jun 10;7:811. doi: 10.3389/fpls.2016.00811 (PMC4901065; doi:10.3389/fpls.2016.00811)

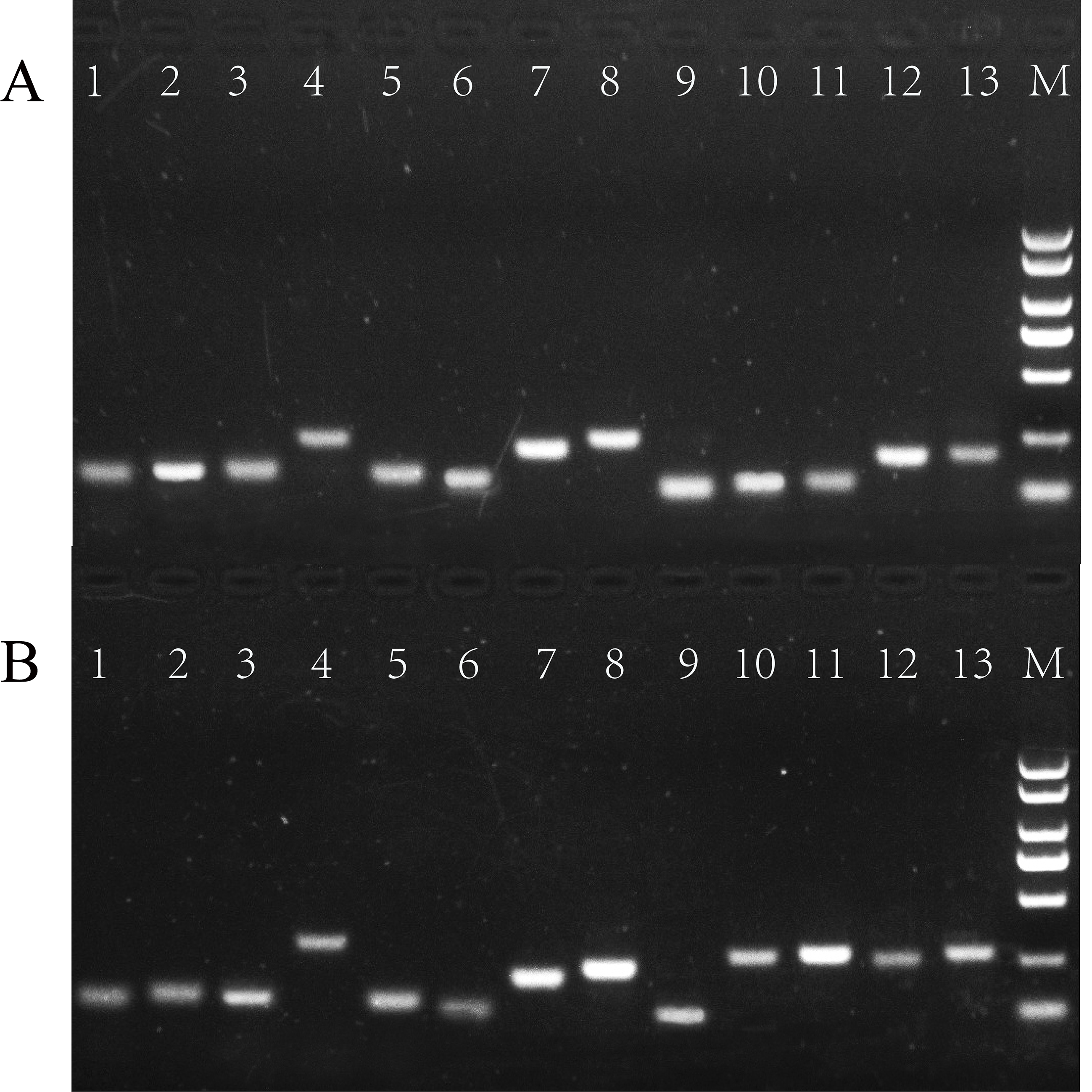

Supplement: Figure S1 — Amplification results of candidate genes with cDNA and genomic DNA of non-heading Chinese cabbage, respectively. Lane 1 ACT7, 2 EF1α, 3TUB4, 4 GAPDH, 5 CYP, 6 DNAJ, 7 HIS, 8 TUA5, 9 ACP, 10 UKN1, 11 SKIP16, 12 CAC, 13 PP2A. (A) The amplification results of 13 candidate genes with cDNA as templates. (B) The amplification results of 13 candidate genes with genomic DNA as templates. M: DL 2000 Plus DNA Marker (from up to low, 2000 bp, 1500 bp, 1000 bp, 750 bp, 500 bp, 250 bp, 100 bp). [file Image1.TIF]

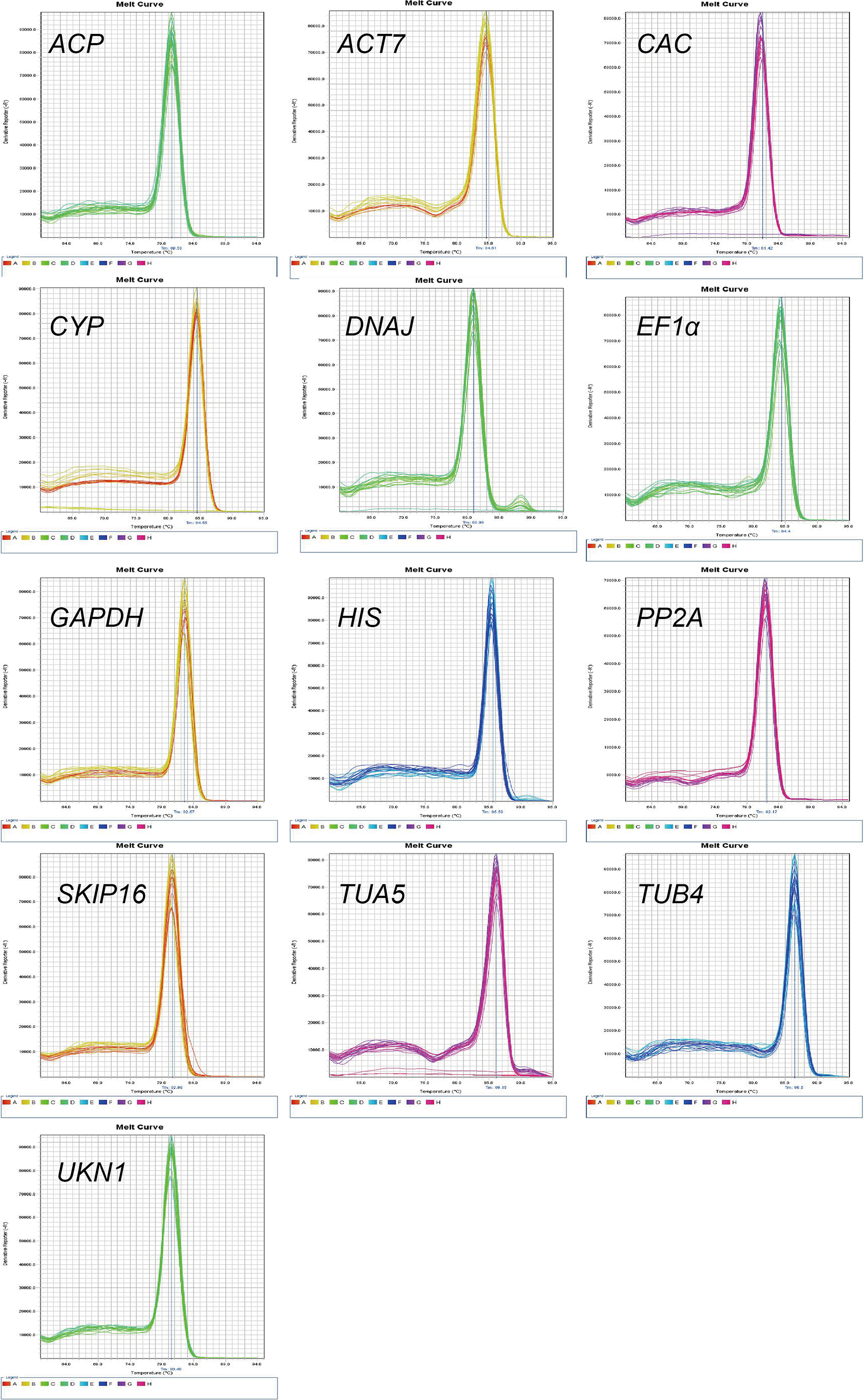

Supplement: Figure S2 — Melting curve analysis of 13 reference genes with the single peak. [file Image2.TIF]
