# Supplementary material for: Identification and Validation of Reference Genes for RT-qPCR Analysis in Non-Heading Chinese Cabbage Flowers
Source: Front Plant Sci. 2016 Jun 10;7:811. doi: 10.3389/fpls.2016.00811 (PMC4901065; doi:10.3389/fpls.2016.00811)
Supplement: Table S1 — Raw Cq values in non-heading Chinese cabbage. FD, different floral development stages; SD, different stigma development stages; FT, different floral tissues; PS, pollinated stigmas. [file DataSheet1.docx]

**Table S1. Raw Cq values in non-heading Chinese cabbage.**

|  | *ACT7* | *EF1α* | *TUB4* | *GAPDH* | *CYP* | *DNAJ* | *HIS* | *TUA5* | *ACP* | *UKN1* | *SKIP16* | *CAC* | *PP2A* |
| --- | --- | --- | --- | --- | --- | --- | --- | --- | --- | --- | --- | --- | --- |
| FD_I_1-1 | 21.46068 | 17.3408 | 20.64515 | 20.90558 | 28.96214 | 23.23541 | 23.25827 | 19.43034 | 20.21536 | 23.47469 | 24.92644 | 22.71609 | 22.27001 |
| FD_I_1-2 | 21.21907 | 17.33732 | 20.5645 | 20.88576 | 28.9267 | 23.15073 | 23.05846 | 19.18834 | 20.17758 | 23.40133 | 24.70062 | 22.63644 | 22.25156 |
| FD_I_1-3 | 21.40164 | 17.29565 | 20.61703 | 20.86136 | 28.98351 | 23.03927 | 23.14741 | 19.17314 | 20.19037 | 23.7171 | 24.76177 | 22.59727 | 22.26129 |
| FD_I_1-4 | 20.85816 | 17.2416 | 20.03811 | 20.38837 | 28.23814 | 22.95738 | 22.93269 | 18.96665 | 19.44279 | 23.01221 | 25.55922 | 22.45197 | 22.08232 |
| FD_I_1-5 | 20.73636 | 17.20158 | 20.10158 | 20.36179 | 28.33216 | 22.88246 | 22.97987 | 19.05111 | 19.23892 | 23.30783 | 25.62285 | 22.41487 | 22.0882 |
| FD_I_1-6 | 20.84097 | 17.14584 | 20.1134 | 20.40545 | 28.26559 | 22.77224 | 23.08624 | 19.30533 | 19.47598 | 23.08959 | 25.48389 | 22.53355 | 22.09687 |
| FD_I_1-7 | 21.67548 | 18.1093 | 21.52739 | 21.98679 | 29.59191 | 22.9739 | 23.58728 | 19.59942 | 20.369 | 23.78628 | 24.90595 | 22.77209 | 22.39342 |
| FD_I_1-8 | 21.65883 | 18.06224 | 21.66936 | 21.97174 | 29.70897 | 23.16928 | 23.67765 | 19.58662 | 20.38452 | 24.05833 | 24.96365 | 22.73182 | 22.4061 |
| FD_I_1-9 | 21.81882 | 18.11168 | 21.51157 | 22.01497 | 29.63373 | 23.08529 | 23.80369 | 19.85099 | 20.40769 | 23.85559 | 25.26857 | 22.85061 | 22.41458 |
| FD_I_2-1 | 19.42883 | 16.86661 | 18.5773 | 18.14306 | 28.20273 | 22.88656 | 21.77811 | 17.26321 | 20.34487 | 22.12815 | 24.68191 | 21.79511 | 21.17732 |
| FD_I_2-2 | 19.47349 | 16.83923 | 18.7364 | 18.13928 | 27.98063 | 22.89928 | 21.87424 | 17.2665 | 20.42056 | 22.23267 | 24.84128 | 21.90245 | 20.94726 |
| FD_I_2-3 | 19.63552 | 16.78597 | 18.65737 | 18.09649 | 28.30516 | 22.94466 | 21.81314 | 17.24467 | 20.3579 | 22.28317 | 24.54084 | 21.90206 | 21.12274 |
| FD_I_2-4 | 20.66106 | 17.84636 | 19.54576 | 19.77165 | 29.56687 | 23.16055 | 22.41203 | 17.80022 | 21.3053 | 22.89655 | 24.96331 | 22.06002 | 21.49806 |
| FD_I_2-5 | 20.66626 | 17.7586 | 19.64835 | 19.74183 | 29.65693 | 23.21519 | 22.45466 | 17.78272 | 21.29658 | 22.98364 | 24.88844 | 22.1748 | 21.44308 |
| FD_I_2-6 | 20.59108 | 17.81769 | 19.71532 | 19.77566 | 29.32894 | 23.16749 | 22.51751 | 17.80541 | 21.36043 | 22.94133 | 25.12398 | 22.17249 | 21.21803 |
| FD_I_2-7 | 20.40008 | 17.00266 | 19.04008 | 18.64117 | 28.89761 | 23.09439 | 21.94784 | 17.38637 | 21.10857 | 22.51434 | 24.82061 | 21.97546 | 21.35196 |
| FD_I_2-8 | 20.33559 | 16.97541 | 19.19917 | 18.64133 | 28.66977 | 23.10203 | 21.99543 | 17.39001 | 21.16696 | 22.62116 | 24.92704 | 22.03904 | 21.07568 |
| FD_I_2-9 | 20.40663 | 16.92193 | 19.12849 | 18.60238 | 28.9441 | 23.14955 | 21.93245 | 17.36787 | 21.10399 | 22.67091 | 24.68227 | 22.04165 | 21.29715 |
| FD_I_3-1 | 20.86196 | 17.41399 | 19.78473 | 21.74754 | 26.52337 | 23.0032 | 22.34327 | 18.94695 | 27.50717 | 23.5037 | 23.8975 | 22.28662 | 21.92674 |
| FD_I_3-2 | 20.94499 | 17.48301 | 19.78247 | 21.84013 | 26.59082 | 22.94569 | 22.38903 | 18.94713 | 27.45403 | 23.462 | 23.77188 | 21.95844 | 22.06676 |
| FD_I_3-3 | 20.82443 | 17.335 | 19.7815 | 21.67574 | 26.55549 | 22.99183 | 22.31588 | 18.93091 | 27.34869 | 23.38968 | 23.72895 | 22.28308 | 21.80941 |
| FD_I_3-4 | 20.05054 | 16.53022 | 18.80075 | 20.23495 | 25.23866 | 22.64167 | 21.74298 | 18.45445 | 26.50739 | 22.77374 | 23.42213 | 21.69113 | 21.78988 |
| FD_I_3-5 | 19.98648 | 16.45743 | 18.80624 | 20.11598 | 25.17884 | 22.74135 | 21.68904 | 18.45019 | 26.55259 | 22.80341 | 23.55426 | 21.97022 | 21.61209 |
| FD_I_3-6 | 19.9418 | 16.38154 | 18.79277 | 20.0988 | 25.21279 | 22.73488 | 21.67117 | 18.42862 | 26.39935 | 22.70642 | 23.38863 | 21.96649 | 21.4871 |
| FD_I_3-7 | 20.60493 | 16.65417 | 19.27157 | 20.61287 | 25.866 | 22.97939 | 21.86338 | 18.57655 | 27.31033 | 23.12923 | 23.69405 | 22.15174 | 21.78381 |
| FD_I_3-8 | 20.56567 | 16.57856 | 19.26387 | 20.5786 | 25.89766 | 22.9698 | 21.84328 | 18.55537 | 27.15327 | 23.02415 | 23.52701 | 22.14812 | 21.6629 |
| FD_I_3-9 | 20.68305 | 16.72634 | 19.26732 | 20.72361 | 25.92797 | 22.88022 | 21.91545 | 18.58004 | 27.25809 | 23.09341 | 23.56413 | 21.87374 | 21.96459 |
| FD_I_4-1 | 20.8688 | 17.83899 | 18.96118 | 21.1649 | 28.78856 | 23.12049 | 22.20569 | 17.25321 | 27.28139 | 23.13522 | 24.47252 | 21.70916 | 21.55565 |
| FD_I_4-2 | 20.73598 | 17.85223 | 18.94023 | 21.04551 | 28.83482 | 23.12149 | 22.13528 | 17.0264 | 27.20139 | 22.92036 | 23.97866 | 21.9589 | 21.98695 |
| FD_I_4-3 | 20.83502 | 17.8483 | 18.94206 | 21.03524 | 28.74172 | 23.15216 | 22.14519 | 17.19482 | 27.27598 | 22.53669 | 24.26015 | 22.01415 | 21.93049 |
| FD_I_4-4 | 21.07378 | 18.90332 | 19.39759 | 22.2767 | 29.44991 | 23.1875 | 22.73934 | 17.67358 | 27.47764 | 23.50389 | 24.68618 | 21.84416 | 21.70374 |
| FD_I_4-5 | 20.9327 | 18.89006 | 19.374 | 22.14508 | 29.62142 | 23.18756 | 22.65487 | 17.44084 | 27.5968 | 23.27883 | 24.12159 | 22.04653 | 22.08574 |
| FD_I_4-6 | 21.03649 | 18.88848 | 19.38035 | 22.12919 | 29.40516 | 23.21666 | 22.67673 | 17.6154 | 27.47318 | 22.91031 | 24.46348 | 22.14519 | 22.07763 |
| FD_I_4-7 | 20.21581 | 17.64128 | 18.46782 | 20.72613 | 28.10246 | 22.90334 | 22.03654 | 17.12823 | 26.53684 | 22.81647 | 24.32745 | 21.52612 | 21.37799 |
| FD_I_4-8 | 20.12356 | 17.66157 | 18.43645 | 20.6126 | 28.17049 | 22.90002 | 21.96912 | 16.95222 | 26.65393 | 22.60164 | 23.84038 | 21.7802 | 21.81639 |
| FD_I_4-9 | 20.19997 | 17.6579 | 18.43278 | 20.61046 | 28.05093 | 22.94149 | 21.9773 | 17.06981 | 26.52098 | 22.15457 | 24.12125 | 21.87833 | 21.80016 |
| FD_C_1-1 | 19.15211 | 17.16906 | 20.12205 | 21.04558 | 20.31732 | 22.26002 | 23.03059 | 18.32085 | 18.69061 | 22.12757 | 23.79609 | 21.97783 | 21.36892 |
| FD_C_1-2 | 19.13511 | 17.15643 | 20.04144 | 20.9804 | 20.37422 | 22.35644 | 23.03345 | 18.13407 | 18.77928 | 22.0619 | 23.90538 | 21.95229 | 21.44766 |
| FD_C_1-3 | 19.14367 | 17.25113 | 20.08969 | 21.10763 | 20.3454 | 22.29839 | 22.97316 | 18.25939 | 18.81254 | 22.04451 | 23.81716 | 21.96152 | 21.39845 |
| FD_C_1-4 | 18.91659 | 16.41335 | 19.66084 | 19.92776 | 19.67114 | 22.19414 | 22.52571 | 17.92513 | 18.49737 | 21.82701 | 23.59356 | 21.89761 | 21.23123 |
| FD_C_1-5 | 18.95749 | 16.42156 | 19.57406 | 19.87306 | 19.72951 | 22.29087 | 22.52553 | 17.79001 | 18.58809 | 21.7662 | 23.70211 | 21.8136 | 21.3075 |
| FD_C_1-6 | 18.9675 | 16.512 | 19.62681 | 19.9254 | 19.70142 | 22.2316 | 22.45813 | 17.91221 | 18.62059 | 21.74881 | 23.61265 | 21.8788 | 21.25949 |
| FD_C_1-7 | 18.37251 | 16.41966 | 19.13857 | 19.42898 | 18.99647 | 21.98721 | 22.34863 | 17.86311 | 17.74655 | 21.44732 | 23.45475 | 21.71201 | 21.06652 |
| FD_C_1-8 | 18.3479 | 16.23257 | 19.05169 | 19.37943 | 19.0557 | 22.05359 | 22.34747 | 17.6665 | 17.84676 | 21.39125 | 23.56246 | 21.6258 | 21.14012 |
| FD_C_1-9 | 18.3625 | 16.32075 | 19.1044 | 19.48043 | 19.02831 | 21.98935 | 22.27957 | 17.78647 | 17.87644 | 21.37459 | 23.47233 | 21.68882 | 21.09353 |
| FD_C_2-1 | 19.43884 | 17.14409 | 19.88522 | 19.63833 | 22.32554 | 23.34599 | 23.12815 | 18.13313 | 19.93014 | 22.26517 | 24.69336 | 22.92332 | 22.32719 |
| FD_C_2-2 | 19.56838 | 17.22108 | 19.89536 | 19.67582 | 22.27755 | 23.36861 | 23.08261 | 18.13526 | 20.02115 | 22.33486 | 24.85651 | 22.74038 | 22.48845 |
| FD_C_2-3 | 19.58511 | 17.21724 | 19.84959 | 19.54938 | 22.4082 | 23.32071 | 22.98997 | 18.01357 | 19.97767 | 22.41615 | 24.63233 | 22.68812 | 22.26976 |
| FD_C_2-4 | 18.87579 | 16.95388 | 19.3661 | 19.13363 | 21.69257 | 23.10681 | 22.95806 | 18.01176 | 19.19473 | 21.94334 | 24.55417 | 22.78104 | 22.157 |
| FD_C_2-5 | 18.94892 | 17.02781 | 19.36243 | 19.1655 | 21.62533 | 23.12885 | 22.95892 | 18.01355 | 19.27909 | 21.96532 | 24.71578 | 22.54921 | 22.2166 |
| FD_C_2-6 | 18.99209 | 17.02456 | 19.33072 | 19.05106 | 21.76216 | 23.08019 | 22.86516 | 17.94643 | 19.23608 | 22.04127 | 24.494 | 22.49766 | 22.10185 |
| FD_C_2-7 | 19.69019 | 17.96846 | 20.33276 | 20.80677 | 22.98309 | 23.41194 | 23.67008 | 18.54458 | 20.11541 | 22.63114 | 24.89557 | 23.06497 | 22.46927 |
| FD_C_2-8 | 19.8243 | 17.96937 | 20.35265 | 20.85488 | 22.94534 | 23.43466 | 23.63209 | 18.54767 | 20.21017 | 22.69732 | 25.00271 | 22.8818 | 22.43142 |
| FD_C_2-9 | 19.83067 | 17.96887 | 20.2931 | 20.69195 | 23.05708 | 23.38695 | 23.52428 | 18.41751 | 20.1653 | 22.78232 | 24.8335 | 22.82903 | 22.41007 |
| FD_C_3-1 | 17.72837 | 16.67022 | 18.17057 | 18.57197 | 21.67576 | 22.28675 | 21.67893 | 16.0087 | 24.13629 | 21.0234 | 23.51917 | 21.47622 | 21.35817 |
| FD_C_3-2 | 17.94493 | 16.84287 | 18.0438 | 18.54571 | 21.78773 | 22.41088 | 21.79955 | 15.9503 | 24.15047 | 20.97112 | 23.44298 | 21.52684 | 21.37049 |
| FD_C_3-3 | 17.85767 | 16.79525 | 18.05977 | 18.82368 | 21.75846 | 22.42637 | 21.72503 | 16.21317 | 24.21983 | 20.98952 | 23.50228 | 21.69346 | 21.2647 |
| FD_C_3-4 | 18.52689 | 17.60971 | 19.15644 | 20.13327 | 22.95569 | 22.59868 | 22.34681 | 16.53344 | 25.06151 | 21.74653 | 23.86029 | 21.80113 | 21.67128 |
| FD_C_3-5 | 18.6731 | 17.7484 | 19.03031 | 20.50558 | 23.09304 | 22.72903 | 22.37861 | 16.77022 | 25.1428 | 21.66718 | 23.84065 | 21.97342 | 21.57433 |
| FD_C_3-6 | 18.77544 | 17.80359 | 19.03667 | 20.13533 | 23.0747 | 22.71259 | 22.45858 | 16.47585 | 25.0947 | 21.70457 | 23.78676 | 21.85885 | 21.67982 |
| FD_C_3-7 | 18.28155 | 16.86173 | 18.69732 | 19.00677 | 22.30651 | 22.53153 | 21.85727 | 16.12879 | 24.92478 | 21.38933 | 23.65818 | 21.66309 | 21.52923 |
| FD_C_3-8 | 18.42069 | 16.93516 | 18.5755 | 19.28507 | 22.42542 | 22.66377 | 21.89981 | 16.33968 | 24.95648 | 21.31075 | 23.64019 | 21.88803 | 21.43385 |
| FD_C_3-9 | 18.53166 | 16.98507 | 18.57106 | 18.98596 | 22.42307 | 22.64754 | 21.92224 | 16.06989 | 24.95762 | 21.34124 | 23.58288 | 21.71761 | 21.53921 |
| FD_C_4-1 | 19.65007 | 18.21229 | 19.93978 | 21.53703 | 23.1976 | 23.98978 | 23.01626 | 17.41015 | 28.10002 | 22.7468 | 24.63083 | 22.81944 | 22.50377 |
| FD_C_4-2 | 19.63619 | 18.15347 | 19.92612 | 21.39436 | 23.29415 | 23.57334 | 23.05179 | 17.5042 | 28.25058 | 22.69597 | 24.6129 | 22.89082 | 22.50863 |
| FD_C_4-3 | 19.65574 | 18.16294 | 19.88043 | 21.36111 | 23.12687 | 23.43537 | 22.96326 | 17.43755 | 28.25559 | 22.63571 | 24.49335 | 22.92627 | 22.10242 |
| FD_C_4-4 | 19.2584 | 17.97919 | 19.37724 | 20.92289 | 22.48813 | 23.70794 | 22.83548 | 17.31455 | 27.51917 | 22.26704 | 24.35486 | 22.73854 | 21.96138 |
| FD_C_4-5 | 19.23239 | 17.97147 | 19.47469 | 20.94664 | 22.65528 | 23.26622 | 22.92621 | 17.37997 | 27.52155 | 22.32499 | 24.4735 | 22.7015 | 22.34181 |
| FD_C_4-6 | 19.15286 | 18.01749 | 19.48479 | 21.03582 | 22.5644 | 23.12209 | 22.89238 | 17.28584 | 27.35966 | 22.37144 | 24.48809 | 22.62932 | 22.33404 |
| FD_C_4-7 | 19.90019 | 19.95322 | 20.3103 | 22.47568 | 23.8298 | 23.36798 | 23.50038 | 17.84846 | 28.44684 | 22.9291 | 24.69518 | 23.00865 | 22.2408 |
| FD_C_4-8 | 19.88313 | 19.93777 | 20.41823 | 22.5174 | 23.95221 | 23.50704 | 23.60244 | 17.91895 | 28.43887 | 22.9894 | 24.81598 | 22.97745 | 22.64724 |
| FD_C_4-9 | 19.8944 | 19.97222 | 20.43218 | 22.70674 | 23.90275 | 23.91849 | 23.55283 | 17.82435 | 28.29009 | 23.04169 | 24.83917 | 22.95955 | 22.64482 |
| SD_I_1-1 | 20.54465 | 16.79014 | 19.78113 | 19.76533 | 28.05659 | 23.6441 | 21.15892 | 18.31338 | 22.93823 | 23.02349 | 22.58295 | 24.0262 | 23.06085 |
| SD_I_1-2 | 20.23691 | 16.60364 | 19.69579 | 19.82729 | 28.16422 | 23.5542 | 21.21413 | 18.24296 | 22.91485 | 23.15126 | 22.54476 | 24.08451 | 22.93948 |
| SD_I_1-3 | 20.36266 | 16.71997 | 19.82675 | 19.81099 | 28.08187 | 23.61066 | 21.37395 | 18.35229 | 22.84188 | 23.17324 | 22.5885 | 23.99024 | 22.79985 |
| SD_I_1-4 | 19.36542 | 16.30418 | 18.97531 | 19.13656 | 27.59344 | 22.51421 | 20.92781 | 17.55498 | 22.33668 | 22.28635 | 21.53006 | 23.22075 | 22.4057 |
| SD_I_1-5 | 19.49586 | 16.22145 | 19.05034 | 19.20956 | 27.21847 | 22.56551 | 21.02766 | 17.65743 | 22.26151 | 22.26071 | 21.55334 | 23.12186 | 22.25937 |
| SD_I_1-6 | 19.67333 | 16.29645 | 19.02266 | 19.26847 | 27.49209 | 22.60442 | 20.87164 | 17.61813 | 22.40889 | 22.15405 | 21.5595 | 23.15896 | 22.09535 |
| SD_I_1-7 | 20.24441 | 16.61243 | 19.58321 | 19.48834 | 27.83999 | 23.20708 | 21.04282 | 18.10471 | 22.79708 | 22.68755 | 22.29795 | 23.41487 | 22.49969 |
| SD_I_1-8 | 20.06699 | 16.53945 | 19.62402 | 19.43109 | 27.86498 | 23.17192 | 21.25308 | 18.14328 | 22.64727 | 22.8207 | 22.29927 | 23.37821 | 22.66215 |
| SD_I_1-9 | 19.94218 | 16.62326 | 19.49584 | 19.35399 | 27.94468 | 23.11733 | 21.09656 | 18.03727 | 22.721 | 22.82141 | 22.26207 | 23.47702 | 22.4826 |
| SD_I_2-1 | 20.83906 | 17.27451 | 20.42661 | 20.88925 | 29.48896 | 23.88832 | 21.36654 | 19.67444 | 23.07004 | 22.82195 | 22.658 | 23.66357 | 22.90288 |
| SD_I_2-2 | 20.84641 | 17.28452 | 20.46162 | 20.83229 | 29.19981 | 23.87677 | 21.66688 | 19.54349 | 22.97433 | 22.86774 | 22.32637 | 23.16132 | 22.43125 |
| SD_I_2-3 | 20.80591 | 17.29737 | 20.47315 | 20.66458 | 29.45414 | 23.83937 | 21.62029 | 19.65421 | 23.1776 | 22.91196 | 21.84277 | 23.36572 | 22.5477 |
| SD_I_2-4 | 21.0481 | 17.5743 | 20.67094 | 21.08808 | 29.72401 | 24.21407 | 21.74125 | 19.86976 | 23.37224 | 23.30486 | 22.07297 | 24.00829 | 23.15556 |
| SD_I_2-5 | 21.08406 | 17.55022 | 20.62053 | 21.28968 | 29.76245 | 24.26527 | 21.48454 | 19.88599 | 23.26487 | 23.15203 | 22.94173 | 24.27517 | 22.75074 |
| SD_I_2-6 | 21.08481 | 17.55878 | 20.65866 | 21.24508 | 29.46403 | 24.25238 | 21.78625 | 19.75708 | 23.16495 | 23.19583 | 22.60952 | 23.83579 | 22.86707 |
| SD_I_2-7 | 20.22502 | 16.97721 | 19.94509 | 20.60631 | 28.90709 | 23.19695 | 21.44191 | 19.00514 | 22.63746 | 22.3315 | 21.59012 | 22.94672 | 22.51021 |
| SD_I_2-8 | 20.20227 | 16.96581 | 19.91721 | 20.66872 | 29.13719 | 23.20618 | 21.14504 | 19.13797 | 22.73116 | 22.28407 | 21.90895 | 23.40696 | 22.04462 |
| SD_I_2-9 | 20.18314 | 16.98719 | 19.95861 | 20.4372 | 29.10643 | 23.16062 | 21.39275 | 19.10965 | 22.83823 | 22.43275 | 21.02117 | 23.10176 | 22.15646 |
| SD_I_3-1 | 20.50425 | 16.8173 | 19.57083 | 20.61823 | 29.03408 | 23.37772 | 22.23621 | 18.73109 | 25.6927 | 22.6322 | 22.45214 | 22.75091 | 21.91954 |
| SD_I_3-2 | 20.56415 | 16.91815 | 19.63957 | 20.58951 | 28.94494 | 23.35639 | 22.23222 | 18.55475 | 25.95072 | 22.32962 | 22.04617 | 22.62842 | 22.1707 |
| SD_I_3-3 | 20.42108 | 16.77903 | 19.5343 | 20.4881 | 28.76321 | 23.39461 | 22.09805 | 18.88418 | 25.92953 | 22.77983 | 21.92273 | 22.92581 | 22.18772 |
| SD_I_3-4 | 20.81599 | 17.02282 | 19.76896 | 21.03268 | 29.29435 | 23.81872 | 22.3549 | 18.94712 | 25.88728 | 22.96606 | 22.7376 | 23.3616 | 22.21868 |
| SD_I_3-5 | 20.87514 | 17.11851 | 19.83783 | 21.00583 | 29.15459 | 23.79477 | 22.35108 | 18.76656 | 26.09495 | 22.74066 | 22.31977 | 23.2421 | 22.48782 |
| SD_I_3-6 | 20.72458 | 16.98368 | 19.72997 | 20.96289 | 28.97602 | 23.83254 | 22.21668 | 19.04775 | 26.12569 | 23.10578 | 22.20022 | 23.61754 | 22.49704 |
| SD_I_3-7 | 19.90932 | 16.50109 | 19.01022 | 20.39482 | 28.74206 | 22.77123 | 22.01391 | 18.18426 | 25.30621 | 22.10271 | 21.70888 | 22.49146 | 21.59323 |
| SD_I_3-8 | 19.9635 | 16.60288 | 19.07708 | 20.36688 | 28.59538 | 22.74759 | 22.00967 | 18.01908 | 25.54958 | 21.85455 | 21.31346 | 22.36735 | 21.83742 |
| SD_I_3-9 | 19.83883 | 16.46341 | 18.98394 | 20.26752 | 28.4152 | 22.78307 | 21.92078 | 18.31853 | 25.58054 | 22.24935 | 21.17026 | 22.71098 | 21.86662 |
| SD_I_4-1 | 20.51654 | 16.97694 | 20.28974 | 20.88084 | 27.88956 | 23.93061 | 22.06704 | 19.38822 | 26.35808 | 22.52391 | 21.60977 | 22.84777 | 22.51337 |
| SD_I_4-2 | 20.54751 | 16.91629 | 20.26702 | 20.93259 | 27.80457 | 23.87676 | 21.93766 | 19.44545 | 26.02055 | 22.72882 | 21.4927 | 22.74207 | 22.66655 |
| SD_I_4-3 | 20.62022 | 16.89166 | 20.23884 | 20.91503 | 27.65356 | 23.92572 | 21.96067 | 19.28616 | 26.18261 | 22.53858 | 21.53132 | 22.66095 | 22.34705 |
| SD_I_4-4 | 19.92677 | 16.66003 | 19.78054 | 20.66223 | 27.53765 | 23.25476 | 21.89059 | 18.89345 | 25.67629 | 21.99554 | 20.84389 | 22.58682 | 22.8424 |
| SD_I_4-5 | 20.00668 | 16.57098 | 19.72366 | 20.70359 | 27.30857 | 23.31319 | 21.73849 | 18.79641 | 25.83856 | 22.01766 | 20.78764 | 22.40914 | 22.65734 |
| SD_I_4-6 | 19.97932 | 16.67592 | 19.76521 | 20.77797 | 27.45484 | 23.1847 | 21.70885 | 18.94307 | 25.9727 | 22.20533 | 20.73836 | 22.48477 | 22.92928 |
| SD_I_4-7 | 20.92245 | 17.09862 | 20.43368 | 21.29622 | 27.91894 | 24.35161 | 22.02929 | 19.49777 | 26.3783 | 22.9339 | 21.81226 | 23.24848 | 21.96818 |
| SD_I_4-8 | 20.82619 | 17.1782 | 20.48461 | 21.27854 | 28.10532 | 24.30129 | 22.18454 | 19.60084 | 26.21498 | 22.92669 | 21.9042 | 23.46409 | 22.11103 |
| SD_I_4-9 | 20.84569 | 17.17431 | 20.46055 | 21.3815 | 28.02035 | 24.26522 | 22.00777 | 19.66529 | 26.55506 | 23.05131 | 21.77865 | 23.34707 | 22.27569 |
| SD_C_1-1 | 17.87945 | 16.34254 | 19.24694 | 19.56053 | 21.59973 | 22.53359 | 21.44764 | 17.90807 | 22.87879 | 21.63649 | 21.51968 | 23.01474 | 22.74837 |
| SD_C_1-2 | 17.98281 | 16.38912 | 19.25933 | 19.49344 | 21.69493 | 22.73541 | 21.48685 | 17.85431 | 22.82669 | 21.83947 | 21.57711 | 22.95188 | 22.74846 |
| SD_C_1-3 | 18.16585 | 16.54785 | 19.29964 | 19.60763 | 21.65848 | 22.5595 | 21.26762 | 17.98318 | 22.86304 | 21.82005 | 21.61197 | 23.14173 | 22.60454 |
| SD_C_1-4 | 18.44436 | 16.65479 | 19.82671 | 19.7969 | 21.94202 | 23.12654 | 21.67992 | 18.40257 | 23.21554 | 22.0872 | 22.24168 | 23.27755 | 22.24834 |
| SD_C_1-5 | 18.59676 | 16.69653 | 19.83416 | 19.72639 | 21.98391 | 23.3372 | 21.71745 | 18.34242 | 23.15979 | 22.29743 | 22.29866 | 23.16492 | 22.2429 |
| SD_C_1-6 | 18.7762 | 16.8561 | 19.87896 | 19.84683 | 21.94714 | 23.15517 | 21.49534 | 18.4841 | 23.19627 | 22.27513 | 22.33977 | 23.40938 | 22.11011 |
| SD_C_1-7 | 18.74399 | 16.92831 | 19.97613 | 20.2524 | 22.14627 | 23.55176 | 21.80359 | 18.61592 | 23.40809 | 22.46198 | 22.51733 | 23.92813 | 22.95185 |
| SD_C_1-8 | 19.00827 | 17.04876 | 20.02501 | 20.30901 | 22.19893 | 23.58004 | 21.61669 | 18.70062 | 23.38573 | 22.66001 | 22.61818 | 24.06028 | 22.86357 |
| SD_C_1-9 | 18.89715 | 16.96565 | 19.98067 | 20.17488 | 22.23752 | 23.76994 | 21.84006 | 18.55333 | 23.34924 | 22.67162 | 22.5736 | 23.852 | 22.95571 |
| SD_C_2-1 | 18.99709 | 17.08453 | 20.8213 | 21.37471 | 21.99461 | 23.81162 | 21.99474 | 19.41931 | 24.97355 | 22.54 | 22.621 | 23.52826 | 22.91828 |
| SD_C_2-2 | 19.13196 | 17.11993 | 20.76036 | 20.92489 | 21.97101 | 23.82536 | 21.99776 | 19.5428 | 25.17184 | 22.44991 | 22.50207 | 23.47656 | 22.96328 |
| SD_C_2-3 | 19.15894 | 17.0761 | 20.74793 | 21.21465 | 21.95567 | 23.75263 | 21.9974 | 19.33999 | 25.16882 | 22.50265 | 22.58584 | 23.69215 | 22.92083 |
| SD_C_2-4 | 19.28889 | 17.35173 | 20.96505 | 21.88765 | 22.24889 | 24.17472 | 22.111 | 19.63416 | 25.16128 | 22.91844 | 22.89555 | 24.17583 | 22.41182 |
| SD_C_2-5 | 19.42884 | 17.3874 | 20.96244 | 21.41825 | 22.22701 | 24.18739 | 22.11519 | 19.75774 | 25.3658 | 22.83188 | 22.78072 | 24.11636 | 22.45678 |
| SD_C_2-6 | 19.45655 | 17.3424 | 20.94666 | 21.7143 | 22.20875 | 24.12485 | 22.11594 | 19.55491 | 25.35938 | 22.87918 | 22.85847 | 24.3259 | 22.45408 |
| SD_C_2-7 | 18.4418 | 16.84223 | 20.24401 | 21.14264 | 21.70316 | 23.15196 | 21.81933 | 18.92632 | 24.64347 | 22.03276 | 21.8799 | 23.25875 | 23.11856 |
| SD_C_2-8 | 18.57683 | 16.87408 | 20.18628 | 20.74962 | 21.6792 | 23.16629 | 21.81859 | 19.00306 | 24.83059 | 21.97952 | 21.76442 | 23.21078 | 23.16033 |
| SD_C_2-9 | 18.59704 | 16.82941 | 20.18224 | 20.98875 | 21.66654 | 23.08284 | 21.81798 | 18.84636 | 24.83103 | 21.99698 | 21.85048 | 23.426 | 23.18125 |
| SD_C_3-1 | 18.85559 | 16.86354 | 19.53296 | 19.78082 | 21.65526 | 23.38824 | 22.30168 | 18.46619 | 26.47639 | 22.41959 | 22.51497 | 23.11864 | 22.86481 |
| SD_C_3-2 | 18.92952 | 16.76308 | 19.79343 | 19.9848 | 21.64411 | 23.44335 | 22.17837 | 18.92304 | 26.59277 | 22.38892 | 22.60582 | 23.55429 | 22.92722 |
| SD_C_3-3 | 18.92294 | 16.70391 | 19.63106 | 20.26446 | 21.81688 | 23.0478 | 21.4955 | 18.2253 | 26.80478 | 22.34799 | 22.45657 | 23.11529 | 22.87261 |
| SD_C_3-4 | 18.24131 | 16.55933 | 18.9676 | 19.55203 | 21.31588 | 22.78772 | 22.07416 | 17.96281 | 26.09433 | 21.95222 | 21.77792 | 22.90667 | 22.34871 |
| SD_C_3-5 | 18.30543 | 16.45657 | 19.21993 | 20.04378 | 21.3034 | 22.83945 | 21.957 | 18.38642 | 26.20937 | 21.91935 | 21.86005 | 23.28163 | 22.42373 |
| SD_C_3-6 | 18.35265 | 16.39569 | 19.06225 | 20.03648 | 21.47435 | 22.45376 | 21.26516 | 17.71474 | 26.41219 | 21.88188 | 21.71672 | 22.90237 | 22.37162 |
| SD_C_3-7 | 19.08777 | 17.05986 | 19.73316 | 20.21628 | 21.91547 | 23.81401 | 22.42338 | 18.68314 | 26.66918 | 22.79053 | 22.79026 | 23.80213 | 23.07244 |
| SD_C_3-8 | 19.16493 | 16.96058 | 19.94121 | 20.68545 | 21.90582 | 23.86824 | 22.29665 | 19.14766 | 26.78576 | 22.76427 | 22.88586 | 24.21238 | 23.12371 |
| SD_C_3-9 | 19.21632 | 16.97322 | 19.832 | 20.76595 | 22.02576 | 23.46675 | 21.61839 | 18.44921 | 26.95188 | 22.72098 | 22.73528 | 23.80513 | 23.06791 |
| SD_C_4-1 | 17.93779 | 16.24263 | 19.38234 | 20.70409 | 20.63679 | 22.82975 | 21.74788 | 18.28859 | 26.11864 | 21.58658 | 21.29471 | 22.44672 | 21.99393 |
| SD_C_4-2 | 18.06659 | 16.00384 | 19.40645 | 20.57369 | 20.504 | 22.62128 | 21.67803 | 18.27838 | 25.98174 | 21.56211 | 21.36624 | 22.63751 | 21.99494 |
| SD_C_4-3 | 18.08583 | 16.23688 | 19.65567 | 20.64431 | 20.36469 | 22.83149 | 21.53971 | 18.2571 | 25.99142 | 21.4576 | 21.19625 | 22.51308 | 21.73042 |
| SD_C_4-4 | 19.21914 | 16.81405 | 20.09207 | 21.3733 | 21.18611 | 23.87762 | 22.05062 | 18.99475 | 26.69389 | 22.40549 | 22.28622 | 23.33415 | 22.75113 |
| SD_C_4-5 | 19.32493 | 16.56334 | 20.11986 | 21.22163 | 21.04393 | 23.63409 | 21.98295 | 18.97618 | 26.54584 | 22.36457 | 22.40278 | 23.53815 | 22.75825 |
| SD_C_4-6 | 19.27376 | 16.82013 | 20.33057 | 21.33801 | 20.95986 | 23.86101 | 21.89405 | 18.96168 | 26.55894 | 22.27661 | 22.19573 | 23.41142 | 22.43213 |
| SD_C_4-7 | 18.51477 | 16.54605 | 19.95493 | 20.93865 | 20.93062 | 23.44331 | 21.93233 | 18.83692 | 26.50089 | 22.03653 | 22.01861 | 22.71127 | 22.48984 |
| SD_C_4-8 | 18.68064 | 16.54765 | 20.14257 | 20.88597 | 20.70175 | 23.43403 | 21.77115 | 18.80238 | 26.36811 | 21.96194 | 21.97961 | 22.78017 | 22.49544 |
| SD_C_4-9 | 18.68095 | 16.30109 | 19.92687 | 20.80189 | 20.84166 | 23.21518 | 21.91228 | 18.81986 | 26.35635 | 22.00535 | 22.11895 | 22.90472 | 22.17405 |
| FT_I_1-1 | 20.68466 | 17.57858 | 20.26012 | 21.84401 | 26.57056 | 24.24892 | 22.51953 | 19.33444 | 25.57558 | 22.87645 | 23.7036 | 24.47351 | 23.40348 |
| FT_I_1-2 | 20.71465 | 17.60881 | 20.14441 | 21.77551 | 26.60664 | 24.25295 | 22.26909 | 19.17918 | 25.67012 | 22.98514 | 23.71679 | 24.75149 | 23.30035 |
| FT_I_1-3 | 20.70923 | 17.4538 | 20.18291 | 21.76893 | 26.70657 | 24.28365 | 22.44243 | 19.18697 | 25.61965 | 23.02369 | 23.77247 | 24.73802 | 23.26525 |
| FT_I_1-4 | 19.76343 | 16.13079 | 19.20231 | 20.92537 | 25.72021 | 23.40056 | 21.30005 | 18.13826 | 24.449 | 21.9144 | 21.678 | 23.37612 | 22.25081 |
| FT_I_1-5 | 19.74838 | 16.24285 | 19.25844 | 20.95195 | 25.59447 | 23.35988 | 21.35773 | 18.28921 | 24.42225 | 21.7042 | 22.01648 | 23.12755 | 22.34395 |
| FT_I_1-6 | 19.7617 | 16.28212 | 19.151 | 20.92737 | 25.62207 | 23.37192 | 21.13612 | 18.13367 | 24.50289 | 21.86151 | 21.73494 | 23.38534 | 22.27852 |
| FT_I_1-7 | 20.2605 | 16.6928 | 19.58104 | 21.17077 | 26.2207 | 23.94916 | 21.93392 | 18.76737 | 25.29557 | 22.53526 | 22.65605 | 24.25705 | 22.88852 |
| FT_I_1-8 | 20.23922 | 16.65843 | 19.69454 | 21.24485 | 26.18959 | 23.94081 | 22.17027 | 18.92412 | 25.20637 | 22.36215 | 22.93361 | 23.99322 | 22.92166 |
| FT_I_1-9 | 20.25924 | 16.54344 | 19.62953 | 21.16833 | 26.32011 | 23.91699 | 22.10073 | 18.77289 | 25.24725 | 22.58377 | 22.60645 | 24.24597 | 22.85731 |
| FT_I_2-1 | 23.06803 | 20.83391 | 18.50557 | 23.33531 | 30.95977 | 25.17035 | 21.53862 | 17.49375 | 29.99617 | 23.35393 | 23.96542 | 27.14039 | 23.8003 |
| FT_I_2-2 | 23.0876 | 20.82929 | 18.56116 | 23.29065 | 30.96686 | 25.07471 | 21.48608 | 17.22622 | 29.97553 | 23.02778 | 23.83585 | 27.04479 | 23.85061 |
| FT_I_2-3 | 23.16355 | 20.91098 | 18.63371 | 23.35662 | 30.96595 | 25.1978 | 21.34183 | 17.33823 | 30.03863 | 22.98901 | 23.71727 | 27.25976 | 23.96348 |
| FT_I_2-4 | 22.12791 | 19.47588 | 17.57899 | 22.45716 | 29.95057 | 24.17474 | 20.36002 | 16.18864 | 28.82251 | 22.55548 | 22.50746 | 25.70532 | 22.82681 |
| FT_I_2-5 | 22.10601 | 19.46217 | 17.50443 | 22.48544 | 29.95076 | 24.2734 | 20.39218 | 16.44865 | 28.8613 | 22.55213 | 22.60044 | 25.81705 | 22.7544 |
| FT_I_2-6 | 22.1982 | 19.54923 | 17.64191 | 22.51258 | 29.96254 | 24.29705 | 20.20217 | 16.29656 | 28.8934 | 22.61569 | 22.36759 | 25.90958 | 22.91664 |
| FT_I_2-7 | 22.69091 | 19.89473 | 17.95289 | 22.7621 | 30.57035 | 24.75396 | 21.14736 | 16.8198 | 29.6445 | 23.21288 | 23.41367 | 26.60239 | 23.37712 |
| FT_I_2-8 | 22.66879 | 19.8858 | 17.94086 | 22.79284 | 30.56787 | 24.85375 | 21.19215 | 17.02495 | 29.67634 | 23.20177 | 23.5305 | 26.71312 | 23.31945 |
| FT_I_2-9 | 22.76521 | 19.91906 | 18.01577 | 22.81919 | 30.62558 | 24.88002 | 21.00219 | 16.92953 | 29.71514 | 23.27097 | 23.28598 | 26.82571 | 23.48103 |
| FT_I_3-1 | 21.46156 | 18.85433 | 20.0537 | 21.54479 | 30.47464 | 25.42178 | 23.05783 | 17.76247 | 27.24771 | 23.78861 | 23.26472 | 26.30971 | 23.66189 |
| FT_I_3-2 | 21.48857 | 18.81812 | 20.12161 | 21.3049 | 30.50396 | 25.40376 | 22.98874 | 17.75963 | 27.1888 | 23.66331 | 23.38803 | 25.91309 | 23.64444 |
| FT_I_3-3 | 21.48991 | 18.73811 | 20.18754 | 21.22764 | 30.36003 | 25.55052 | 22.93301 | 18.119 | 26.61422 | 23.08362 | 23.71797 | 25.97561 | 23.47884 |
| FT_I_3-4 | 20.9755 | 18.32095 | 19.81404 | 20.93334 | 29.75477 | 24.97906 | 22.07935 | 17.53824 | 25.7777 | 22.42894 | 22.78037 | 25.09402 | 22.89147 |
| FT_I_3-5 | 20.9689 | 18.40157 | 19.74934 | 21.0032 | 29.88685 | 24.88416 | 22.18586 | 17.12689 | 26.37412 | 22.98602 | 22.46533 | 24.97876 | 23.03667 |
| FT_I_3-6 | 20.94472 | 18.43771 | 19.6836 | 21.23946 | 29.85961 | 24.8998 | 22.26068 | 17.1305 | 26.45656 | 23.09511 | 22.346 | 25.42566 | 23.05457 |
| FT_I_3-7 | 21.9207 | 19.79119 | 20.67317 | 22.10346 | 30.86625 | 25.80459 | 23.39694 | 18.17591 | 27.62008 | 24.22242 | 23.69471 | 26.80586 | 24.07558 |
| FT_I_3-8 | 21.95029 | 19.74922 | 20.74347 | 21.92075 | 30.90049 | 25.78521 | 23.32908 | 18.17355 | 27.57997 | 24.09038 | 23.82097 | 26.34238 | 24.06162 |
| FT_I_3-9 | 21.94953 | 19.66506 | 20.81068 | 21.82692 | 30.74966 | 25.93327 | 23.2211 | 18.59368 | 26.94193 | 23.59149 | 24.08211 | 26.45946 | 23.96628 |
| FT_I_4-1 | 20.88599 | 19.17458 | 19.40121 | 22.36694 | 29.30717 | 24.21607 | 23.34754 | 16.65326 | 27.80947 | 22.92501 | 22.94664 | 24.93939 | 22.59809 |
| FT_I_4-2 | 20.87859 | 19.12451 | 19.27192 | 22.12399 | 28.93104 | 24.28364 | 23.56307 | 16.35539 | 27.76018 | 22.60646 | 22.75812 | 24.83244 | 22.5654 |
| FT_I_4-3 | 20.93073 | 19.13599 | 19.4347 | 22.10512 | 29.32192 | 24.27193 | 23.52483 | 15.93745 | 27.89676 | 22.6722 | 22.88442 | 24.57945 | 22.30649 |
| FT_I_4-4 | 21.32109 | 20.04248 | 19.9943 | 22.6922 | 29.72475 | 24.65118 | 23.87094 | 16.40543 | 28.21982 | 23.1017 | 23.25095 | 25.01067 | 22.78316 |
| FT_I_4-5 | 21.26638 | 20.03177 | 19.88258 | 22.70874 | 29.30566 | 24.6607 | 23.90698 | 16.82635 | 28.07981 | 23.04908 | 23.12112 | 25.25213 | 22.98283 |
| FT_I_4-6 | 21.28203 | 20.09423 | 19.96285 | 22.97344 | 29.70722 | 24.59364 | 23.6876 | 17.09352 | 28.127 | 23.44669 | 23.36712 | 25.36508 | 23.01377 |
| FT_I_4-7 | 20.32051 | 18.79934 | 18.98034 | 22.06706 | 28.68763 | 23.70593 | 22.553 | 16.00203 | 26.98676 | 22.25153 | 22.0325 | 24.01241 | 21.99257 |
| FT_I_4-8 | 20.32017 | 18.75191 | 18.89962 | 21.8818 | 28.32152 | 23.77342 | 22.75628 | 15.77768 | 26.93776 | 21.93442 | 21.83764 | 23.94167 | 21.99065 |
| FT_I_4-9 | 20.3724 | 18.76145 | 19.0124 | 21.8647 | 28.69954 | 23.76108 | 22.72768 | 15.35051 | 27.04572 | 21.99656 | 21.93703 | 23.70331 | 21.75138 |
| FT_I_5-1 | 19.50253 | 16.62998 | 18.93356 | 19.85951 | 27.22345 | 23.2373 | 22.0881 | 16.96246 | 27.78874 | 22.01201 | 23.5752 | 22.32719 | 22.70435 |
| FT_I_5-2 | 19.40287 | 16.65862 | 18.80018 | 19.48361 | 27.28596 | 23.19489 | 22.06582 | 16.94844 | 27.75018 | 21.63238 | 23.72741 | 22.18777 | 22.85291 |
| FT_I_5-3 | 19.635 | 16.77667 | 18.68336 | 19.33423 | 26.9959 | 23.09426 | 22.00673 | 16.99815 | 27.72263 | 21.77235 | 22.76402 | 22.34112 | 23.02416 |
| FT_I_5-4 | 20.07174 | 17.40768 | 19.75292 | 20.20612 | 27.92981 | 23.95946 | 23.17452 | 17.64602 | 28.16762 | 22.90076 | 24.36124 | 22.75615 | 23.27134 |
| FT_I_5-5 | 19.98059 | 17.42754 | 19.63426 | 19.86359 | 28.00097 | 23.91419 | 23.15926 | 17.58632 | 28.11881 | 22.46587 | 24.52056 | 22.61535 | 23.23065 |
| FT_I_5-6 | 20.20708 | 17.55962 | 19.49336 | 19.71677 | 27.75455 | 23.81359 | 23.10299 | 17.67451 | 28.09391 | 22.60337 | 23.57555 | 22.78292 | 23.09837 |
| FT_I_5-7 | 20.73071 | 17.8646 | 20.04573 | 20.95378 | 28.43717 | 24.38827 | 23.61563 | 18.08816 | 28.71361 | 23.23693 | 24.67434 | 23.01026 | 23.61353 |
| FT_I_5-8 | 20.63685 | 17.87467 | 19.93262 | 20.54663 | 28.51564 | 24.34207 | 23.59613 | 18.02804 | 28.64529 | 22.87223 | 24.8322 | 22.92691 | 23.42108 |
| FT_I_5-9 | 20.86556 | 17.93706 | 19.84738 | 20.39582 | 28.21541 | 24.23753 | 23.54828 | 18.1148 | 28.63464 | 22.94975 | 23.90776 | 23.04489 | 23.70389 |
| FT_C_1-1 | 18.92586 | 16.38228 | 19.11729 | 20.94423 | 21.73259 | 23.88306 | 22.15868 | 18.64603 | 25.95952 | 21.94412 | 22.8525 | 23.88822 | 22.97731 |
| FT_C_1-2 | 18.97188 | 16.29643 | 19.55791 | 20.89411 | 21.9418 | 23.70139 | 21.66914 | 18.26542 | 26.04162 | 21.94742 | 22.87701 | 23.69905 | 22.92211 |
| FT_C_1-3 | 18.8503 | 16.43594 | 19.78914 | 20.95818 | 21.84262 | 23.61374 | 21.83536 | 18.2041 | 26.02389 | 21.87574 | 22.93576 | 23.84694 | 22.96733 |
| FT_C_1-4 | 18.40779 | 15.9358 | 19.13151 | 20.59099 | 21.32739 | 23.12917 | 20.8581 | 17.68893 | 25.23266 | 21.30491 | 21.95978 | 22.80591 | 22.33665 |
| FT_C_1-5 | 18.41614 | 15.9653 | 18.73825 | 20.64125 | 21.11882 | 23.30423 | 21.35812 | 18.02145 | 25.09148 | 21.3043 | 21.91928 | 22.98077 | 22.34178 |
| FT_C_1-6 | 18.2907 | 16.02447 | 19.36073 | 20.64484 | 21.22959 | 23.04203 | 20.98626 | 17.62277 | 25.21152 | 21.19375 | 21.9911 | 22.93693 | 22.33883 |
| FT_C_1-7 | 19.37354 | 17.31702 | 19.73945 | 21.47141 | 22.03395 | 24.19287 | 22.50148 | 19.05317 | 26.28501 | 22.43352 | 23.21433 | 24.30721 | 23.27346 |
| FT_C_1-8 | 19.36772 | 17.24258 | 20.11818 | 21.42167 | 22.24906 | 24.01266 | 21.96804 | 18.73436 | 26.42155 | 22.43733 | 23.23428 | 24.11935 | 23.27482 |
| FT_C_1-9 | 19.24149 | 17.35976 | 20.34147 | 21.50929 | 22.14512 | 23.93283 | 22.12742 | 18.67987 | 26.40487 | 22.30955 | 23.30502 | 24.2727 | 23.25941 |
| FT_C_2-1 | 20.15779 | 18.94842 | 17.35015 | 21.65258 | 24.17711 | 23.71667 | 20.84694 | 15.73573 | 28.72501 | 21.20282 | 21.85378 | 24.15926 | 22.51017 |
| FT_C_2-2 | 19.96146 | 18.84446 | 17.40176 | 21.67934 | 24.00682 | 23.80928 | 20.54835 | 15.83399 | 28.46118 | 21.53541 | 21.80807 | 24.15197 | 22.50245 |
| FT_C_2-3 | 20.14671 | 19.00452 | 17.31176 | 21.56959 | 24.26476 | 23.72976 | 20.78439 | 15.70615 | 28.21655 | 21.3896 | 21.83063 | 24.1664 | 22.63419 |
| FT_C_2-4 | 20.9292 | 20.18319 | 18.36964 | 22.53243 | 24.98046 | 24.70151 | 21.68264 | 16.88603 | 29.66246 | 22.66872 | 23.08781 | 25.50512 | 23.4119 |
| FT_C_2-5 | 21.12864 | 20.2552 | 18.33214 | 22.49722 | 25.11603 | 24.60744 | 21.95041 | 16.79354 | 29.94727 | 22.32503 | 23.14751 | 25.53446 | 23.45994 |
| FT_C_2-6 | 21.10927 | 20.36003 | 18.28838 | 22.41573 | 25.19095 | 24.61768 | 21.93372 | 16.75108 | 29.40479 | 22.51938 | 23.14475 | 25.53029 | 23.56601 |
| FT_C_2-7 | 20.52188 | 19.23609 | 17.82464 | 21.9375 | 24.624 | 24.32117 | 21.33975 | 16.41249 | 29.27393 | 22.17497 | 22.72406 | 25.02245 | 23.0641 |
| FT_C_2-8 | 20.72911 | 19.33103 | 17.77704 | 21.9564 | 24.80626 | 24.22968 | 21.655 | 16.31717 | 29.55329 | 21.88917 | 22.78478 | 25.04428 | 23.09837 |
| FT_C_2-9 | 20.71275 | 19.42624 | 17.737 | 21.87481 | 24.88664 | 24.24068 | 21.58865 | 16.28152 | 29.02519 | 22.02953 | 22.7717 | 25.04465 | 23.20918 |
| FT_C_3-1 | 18.30599 | 16.12749 | 19.04994 | 20.21781 | 20.93099 | 23.234 | 21.53144 | 17.96025 | 28.63357 | 21.96931 | 22.08873 | 22.72438 | 22.37047 |
| FT_C_3-2 | 18.40959 | 16.24121 | 18.97337 | 20.50575 | 21.17691 | 23.32573 | 21.61779 | 18.20477 | 28.76731 | 22.1666 | 22.08461 | 22.92806 | 22.37383 |
| FT_C_3-3 | 18.52083 | 16.03739 | 18.92862 | 20.21431 | 21.02996 | 23.18482 | 21.58966 | 17.97428 | 28.65869 | 22.15192 | 22.21959 | 22.77258 | 22.4447 |
| FT_C_3-4 | 18.87101 | 17.17081 | 19.58736 | 21.09396 | 21.53321 | 23.70189 | 21.97903 | 18.69907 | 29.09445 | 22.65115 | 22.50178 | 23.42043 | 22.7301 |
| FT_C_3-5 | 18.76451 | 17.04444 | 19.66155 | 20.80958 | 21.23237 | 23.60862 | 21.87684 | 18.41748 | 28.96282 | 22.46448 | 22.50234 | 23.1462 | 22.73059 |
| FT_C_3-6 | 18.92772 | 16.96524 | 19.54404 | 20.81273 | 21.38422 | 23.55893 | 21.94021 | 18.43684 | 28.9951 | 22.63951 | 22.64177 | 23.20022 | 22.80377 |
| FT_C_3-7 | 17.80061 | 15.76526 | 18.67803 | 19.98163 | 20.31361 | 22.72032 | 20.72672 | 17.38533 | 27.80645 | 21.3278 | 21.19252 | 21.83981 | 21.79322 |
| FT_C_3-8 | 17.89627 | 15.87375 | 18.5995 | 20.18673 | 20.60076 | 22.81086 | 20.79074 | 17.60965 | 27.92914 | 21.53319 | 21.17996 | 22.0372 | 21.79949 |
| FT_C_3-9 | 17.96031 | 15.67265 | 18.54915 | 19.97869 | 20.45413 | 22.67259 | 20.77811 | 17.3989 | 27.82101 | 21.52144 | 21.31099 | 21.88048 | 21.86758 |
| FT_C_4-1 | 19.20465 | 18.6354 | 19.14122 | 21.06377 | 23.83208 | 23.06256 | 22.82886 | 16.20152 | 26.11981 | 21.06949 | 22.13745 | 23.72293 | 22.02057 |
| FT_C_4-2 | 19.15432 | 18.52353 | 19.01303 | 21.10413 | 23.77141 | 22.83116 | 22.63741 | 16.34368 | 26.16121 | 20.56576 | 21.44981 | 23.72512 | 22.0048 |
| FT_C_4-3 | 19.17728 | 18.62889 | 19.13478 | 21.09815 | 23.91455 | 22.91203 | 22.81562 | 16.27165 | 26.10725 | 20.96074 | 22.1111 | 23.74498 | 22.11167 |
| FT_C_4-4 | 20.17601 | 19.93753 | 20.12961 | 21.95705 | 24.77972 | 23.97527 | 23.94914 | 17.23161 | 27.32054 | 22.18621 | 23.46771 | 25.13395 | 22.95462 |
| FT_C_4-5 | 20.12592 | 19.90303 | 20.12515 | 21.93599 | 24.8531 | 23.80159 | 23.93288 | 17.30083 | 27.31027 | 22.06011 | 23.43188 | 25.13999 | 23.03112 |
| FT_C_4-6 | 20.10223 | 19.84699 | 20.00892 | 21.951 | 24.72398 | 23.72064 | 23.78091 | 17.39768 | 27.35162 | 21.7092 | 22.78462 | 25.06671 | 22.93977 |
| FT_C_4-7 | 19.73761 | 18.99465 | 19.56427 | 21.39149 | 24.50169 | 23.42868 | 23.63307 | 16.89468 | 26.92896 | 21.63206 | 23.01661 | 24.615 | 22.73783 |
| FT_C_4-8 | 19.77666 | 19.00749 | 19.56849 | 21.35982 | 24.42094 | 23.64561 | 23.65071 | 16.82606 | 26.93966 | 21.755 | 23.04767 | 24.60202 | 22.65246 |
| FT_C_4-9 | 19.71136 | 18.92811 | 19.44018 | 21.40126 | 24.36519 | 23.34414 | 23.43631 | 16.92972 | 26.97337 | 21.21113 | 22.35867 | 24.62463 | 22.63655 |
| FT_C_5-1 | 18.52334 | 17.79009 | 19.96299 | 20.09593 | 22.59505 | 23.93694 | 23.15478 | 17.66374 | 28.20194 | 22.53948 | 24.49082 | 22.92371 | 22.73113 |
| FT_C_5-2 | 18.59536 | 17.66095 | 19.92836 | 20.11292 | 22.5892 | 23.9212 | 23.20391 | 17.6937 | 28.22213 | 22.49553 | 24.49183 | 22.95684 | 22.73218 |
| FT_C_5-3 | 18.37227 | 17.98606 | 19.89783 | 19.90541 | 22.55952 | 23.96666 | 23.15741 | 17.5029 | 28.20284 | 22.49091 | 24.49408 | 22.98836 | 22.45707 |
| FT_C_5-4 | 19.09033 | 18.1589 | 20.25548 | 20.86757 | 23.04765 | 24.41687 | 23.59198 | 18.10098 | 28.73934 | 22.94232 | 24.80199 | 23.24427 | 23.51862 |
| FT_C_5-5 | 19.16073 | 18.03408 | 20.28008 | 20.87254 | 23.04342 | 24.34619 | 23.6494 | 18.12165 | 28.74616 | 22.89535 | 24.80688 | 23.26775 | 23.52802 |
| FT_C_5-6 | 18.94175 | 18.42518 | 20.191 | 20.60899 | 23.01227 | 24.44563 | 23.59622 | 17.94974 | 28.73834 | 22.89786 | 24.82128 | 23.29977 | 23.18686 |
| FT_C_5-7 | 17.95648 | 16.99013 | 19.09911 | 19.74994 | 21.89074 | 23.27226 | 22.07083 | 16.99057 | 27.83113 | 21.71327 | 23.70834 | 22.52455 | 23.24687 |
| FT_C_5-8 | 17.99533 | 16.85731 | 19.10332 | 19.77286 | 21.87583 | 23.20398 | 22.09923 | 17.02836 | 27.85706 | 21.65531 | 23.70232 | 22.57345 | 23.2527 |
| FT_C_5-9 | 17.81405 | 17.22176 | 19.0392 | 19.51421 | 21.85626 | 23.302 | 22.05983 | 16.87069 | 27.83673 | 21.64865 | 23.68219 | 22.60749 | 22.91846 |
| PS_I_1-1 | 21.23306 | 16.73554 | 19.77915 | 19.63594 | 29.24189 | 23.95926 | 22.1639 | 19.17679 | 26.41751 | 23.32189 | 25.43314 | 22.92358 | 23.25937 |
| PS_I_1-2 | 21.48781 | 16.91319 | 19.79469 | 19.66022 | 29.4002 | 24.4687 | 22.1799 | 19.05297 | 26.39976 | 23.30993 | 25.34413 | 23.13594 | 23.01718 |
| PS_I_1-3 | 21.42141 | 16.9115 | 19.81738 | 19.65472 | 29.57797 | 23.9749 | 22.08291 | 19.16448 | 26.44729 | 23.34459 | 25.21748 | 23.29311 | 22.43661 |
| PS_I_1-4 | 20.16536 | 16.27747 | 19.41453 | 18.92577 | 28.78999 | 23.52015 | 21.77949 | 18.74056 | 25.94507 | 22.97593 | 25.12179 | 22.66421 | 23.54072 |
| PS_I_1-5 | 20.13346 | 16.45339 | 19.42806 | 18.95633 | 28.95182 | 23.91896 | 21.76871 | 18.6071 | 25.93525 | 22.97308 | 25.03221 | 22.88297 | 23.71429 |
| PS_I_1-6 | 20.26242 | 16.45205 | 19.45886 | 18.94937 | 29.06994 | 23.4738 | 21.69216 | 18.7247 | 25.92622 | 22.94513 | 24.96662 | 22.984 | 23.56929 |
| PS_I_1-7 | 19.58646 | 15.507 | 18.5881 | 18.53246 | 28.02545 | 22.82636 | 20.65496 | 18.06821 | 25.51571 | 22.08134 | 24.3385 | 22.23104 | 23.56932 |
| PS_I_1-8 | 19.56257 | 15.68212 | 18.60478 | 18.56691 | 28.19556 | 23.17161 | 20.59936 | 17.96823 | 25.51367 | 22.0858 | 24.24312 | 22.44637 | 23.42608 |
| PS_I_1-9 | 19.69292 | 15.68081 | 18.64545 | 18.55876 | 28.37025 | 22.78445 | 20.55582 | 18.04784 | 25.55991 | 22.11471 | 24.12915 | 22.60637 | 23.23934 |
| PS_I_2-1 | 20.14446 | 15.97358 | 19.09105 | 18.97581 | 28.61541 | 23.49088 | 21.00052 | 18.65066 | 26.27405 | 22.0409 | 24.28232 | 22.75782 | 23.26805 |
| PS_I_2-2 | 20.252 | 15.72636 | 19.08693 | 19.05206 | 28.91114 | 23.35883 | 20.83973 | 18.54854 | 26.01846 | 22.39499 | 24.54774 | 22.94191 | 22.78384 |
| PS_I_2-3 | 20.18446 | 16.13611 | 18.97973 | 19.19518 | 28.78219 | 23.46341 | 20.86558 | 18.36186 | 26.28583 | 22.07493 | 24.85516 | 22.76003 | 22.90016 |
| PS_I_2-4 | 21.36425 | 17.16642 | 20.2428 | 20.03936 | 29.90721 | 24.67453 | 22.54057 | 19.78469 | 27.16768 | 23.27437 | 25.38051 | 23.4455 | 23.93922 |
| PS_I_2-5 | 21.46853 | 16.9654 | 20.24234 | 20.15056 | 30.14721 | 24.53164 | 22.37848 | 19.68054 | 26.97071 | 23.6267 | 25.67262 | 23.64434 | 23.51821 |
| PS_I_2-6 | 21.42099 | 17.35716 | 20.12068 | 20.29152 | 29.96012 | 24.63697 | 22.3732 | 19.48065 | 27.1862 | 23.31892 | 25.9732 | 23.45874 | 23.63919 |
| PS_I_2-7 | 20.87946 | 16.50544 | 19.95111 | 19.4437 | 29.65485 | 24.03578 | 21.93595 | 19.17548 | 26.43514 | 23.21828 | 25.35479 | 23.32653 | 23.48594 |
| PS_I_2-8 | 20.77435 | 16.78332 | 19.95002 | 19.3269 | 29.35745 | 24.17194 | 22.09946 | 19.27831 | 26.69794 | 22.93555 | 25.07024 | 23.13451 | 23.67642 |
| PS_I_2-9 | 20.82073 | 16.91064 | 19.82273 | 19.58842 | 29.49356 | 24.1399 | 21.93747 | 18.98601 | 26.71338 | 22.91385 | 25.65532 | 23.14265 | 23.30704 |
| PS_I_3-1 | 20.92689 | 16.88957 | 20.137 | 19.694 | 29.52686 | 23.96114 | 22.15184 | 19.75164 | 26.67017 | 23.30189 | 25.92751 | 23.4882 | 22.65376 |
| PS_I_3-2 | 20.89658 | 16.88718 | 20.14271 | 19.67131 | 29.4168 | 23.95707 | 22.03924 | 19.62867 | 26.71952 | 23.14919 | 26.08562 | 23.51049 | 22.91497 |
| PS_I_3-3 | 20.93288 | 16.82727 | 20.04442 | 19.62521 | 29.36863 | 23.91591 | 22.15325 | 19.60784 | 26.54687 | 23.10398 | 25.92385 | 23.36465 | 22.93267 |
| PS_I_3-4 | 21.57603 | 17.26626 | 20.49287 | 20.40366 | 29.98735 | 24.44987 | 22.60052 | 20.19574 | 27.14155 | 23.71861 | 26.235 | 23.81055 | 22.31439 |
| PS_I_3-5 | 21.49387 | 17.26207 | 20.50096 | 20.38123 | 29.95306 | 24.38866 | 22.48074 | 20.07081 | 27.19139 | 23.55736 | 26.39761 | 23.82972 | 22.56836 |
| PS_I_3-6 | 21.51849 | 17.19734 | 20.3944 | 20.32252 | 29.89805 | 24.34334 | 22.59703 | 20.05506 | 27.02323 | 23.51054 | 26.1767 | 23.6831 | 22.59873 |
| PS_I_3-7 | 20.35582 | 16.05575 | 19.31995 | 19.29682 | 28.81487 | 23.28819 | 21.03665 | 19.07125 | 26.24714 | 22.45994 | 25.13943 | 23.05186 | 22.96487 |
| PS_I_3-8 | 20.26182 | 16.05759 | 19.32555 | 19.27586 | 28.68651 | 23.22607 | 20.96031 | 18.95803 | 26.29386 | 22.32187 | 25.3007 | 23.07697 | 23.24477 |
| PS_I_3-9 | 20.31122 | 16.00569 | 19.23728 | 19.23401 | 28.64263 | 23.19365 | 21.06021 | 18.9775 | 26.12565 | 22.27217 | 25.08527 | 22.97864 | 23.25236 |
| PS_C_1-1 | 19.16691 | 16.7605 | 19.93206 | 19.20169 | 22.13501 | 23.94729 | 21.69178 | 18.92197 | 26.35407 | 22.76892 | 25.20689 | 23.53274 | 23.51575 |
| PS_C_1-2 | 19.21483 | 16.70545 | 19.91665 | 19.08496 | 22.2166 | 23.96464 | 21.58308 | 18.88071 | 26.4752 | 22.69956 | 25.29099 | 23.68271 | 23.51584 |
| PS_C_1-3 | 19.33667 | 16.79725 | 20.05489 | 19.22635 | 22.29188 | 23.9094 | 21.46478 | 18.9763 | 26.43571 | 22.87103 | 25.2381 | 23.56232 | 23.36616 |
| PS_C_1-4 | 18.61019 | 15.95915 | 19.04916 | 18.85259 | 21.4287 | 23.25712 | 20.57051 | 18.29942 | 25.9833 | 21.89709 | 24.42457 | 23.08911 | 23.72737 |
| PS_C_1-5 | 18.66153 | 15.89512 | 19.03718 | 18.73842 | 21.51023 | 23.23369 | 20.45857 | 18.20943 | 26.05653 | 21.83562 | 24.50469 | 23.2438 | 23.63556 |
| PS_C_1-6 | 18.78104 | 15.97309 | 19.23083 | 18.87378 | 21.58995 | 23.18456 | 20.34256 | 18.29677 | 26.01909 | 21.98911 | 24.44305 | 23.11808 | 23.73138 |
| PS_C_1-7 | 19.81283 | 17.14527 | 20.23527 | 19.94629 | 22.64685 | 24.44192 | 22.08559 | 19.41168 | 26.89331 | 23.12017 | 25.51944 | 23.86192 | 22.99572 |
| PS_C_1-8 | 19.85553 | 17.0887 | 20.22042 | 19.86739 | 22.73155 | 24.39733 | 21.97688 | 19.30908 | 26.9519 | 23.04955 | 25.60478 | 23.94641 | 22.99006 |
| PS_C_1-9 | 19.92361 | 17.16946 | 20.41355 | 19.98946 | 22.80307 | 24.34003 | 21.92008 | 19.41344 | 26.97245 | 23.20696 | 25.55522 | 23.89236 | 22.85195 |
| PS_C_2-1 | 18.9795 | 16.76135 | 19.61894 | 18.86966 | 21.59169 | 23.64532 | 21.51899 | 18.72873 | 26.36994 | 22.56054 | 24.44428 | 23.36265 | 23.69245 |
| PS_C_2-2 | 18.90161 | 16.69285 | 19.6761 | 18.69446 | 21.75023 | 23.54524 | 21.52732 | 18.74721 | 26.30758 | 22.93523 | 24.50129 | 23.42865 | 23.73925 |
| PS_C_2-3 | 18.90561 | 16.79378 | 19.57695 | 18.72851 | 21.67318 | 23.59856 | 21.21001 | 18.64838 | 26.44236 | 22.79707 | 24.44621 | 23.30861 | 23.69511 |
| PS_C_2-4 | 19.554 | 17.14261 | 19.92985 | 19.60161 | 22.04479 | 24.07942 | 21.96431 | 19.17006 | 26.90825 | 22.91162 | 24.75656 | 23.6895 | 23.16574 |
| PS_C_2-5 | 19.48454 | 17.06671 | 19.98303 | 19.41696 | 22.20075 | 23.9792 | 21.97469 | 19.18718 | 26.85099 | 23.28105 | 24.8162 | 23.76029 | 23.21249 |
| PS_C_2-6 | 19.47566 | 17.17011 | 19.93657 | 19.44527 | 22.11976 | 24.0291 | 21.65099 | 19.08245 | 26.92927 | 23.141 | 24.75769 | 23.63588 | 23.20968 |
| PS_C_2-7 | 18.36868 | 15.96411 | 18.7867 | 18.46318 | 20.88544 | 22.95451 | 20.40461 | 18.04842 | 25.95338 | 21.72688 | 23.66425 | 22.97684 | 23.90075 |
| PS_C_2-8 | 18.2793 | 15.89725 | 18.83944 | 18.29241 | 21.00091 | 22.86545 | 20.42149 | 18.06689 | 25.93593 | 22.04243 | 23.7173 | 22.98287 | 23.94418 |
| PS_C_2-9 | 18.29454 | 15.99064 | 18.75584 | 18.32812 | 20.96634 | 22.91374 | 20.10848 | 17.9853 | 26.0217 | 21.93993 | 23.65864 | 22.91511 | 23.96594 |
| PS_C_3-1 | 19.97513 | 17.60997 | 20.72633 | 20.68078 | 23.22514 | 24.48954 | 23.06349 | 19.95288 | 27.17882 | 23.22086 | 26.05611 | 24.63267 | 23.63684 |
| PS_C_3-2 | 20.10751 | 17.64947 | 20.79081 | 20.92274 | 23.31704 | 24.43675 | 22.9322 | 20.10591 | 26.99491 | 23.1966 | 25.95454 | 24.87453 | 23.70175 |
| PS_C_3-3 | 20.20486 | 17.5611 | 20.69754 | 20.68705 | 23.36702 | 24.49007 | 22.9268 | 19.95844 | 27.14298 | 23.24564 | 25.98356 | 24.66884 | 23.64496 |
| PS_C_3-4 | 20.60612 | 17.98303 | 21.02381 | 21.39858 | 23.72861 | 24.92307 | 23.49813 | 20.44251 | 27.71315 | 23.62715 | 26.36182 | 24.95314 | 23.1001 |
| PS_C_3-5 | 20.85123 | 17.94111 | 20.99884 | 21.38188 | 23.88008 | 24.97591 | 23.35441 | 20.39968 | 27.67931 | 23.65144 | 26.28944 | 24.94225 | 23.12393 |
| PS_C_3-6 | 20.74317 | 18.02768 | 21.08537 | 21.73279 | 23.82556 | 24.92149 | 23.32056 | 20.61991 | 27.52415 | 23.59945 | 26.2043 | 25.14938 | 23.17812 |
| PS_C_3-7 | 19.42043 | 16.82245 | 19.88382 | 20.27624 | 22.53095 | 23.81311 | 21.99627 | 19.32669 | 26.81306 | 22.3861 | 25.28493 | 24.19693 | 23.85218 |
| PS_C_3-8 | 19.55005 | 16.85653 | 19.94881 | 20.57215 | 22.62449 | 23.76606 | 21.80296 | 19.46969 | 26.62574 | 22.3675 | 25.12406 | 24.40978 | 23.9061 |
| PS_C_3-9 | 19.64313 | 16.78043 | 19.85971 | 20.29485 | 22.66773 | 23.81587 | 21.88767 | 19.2751 | 26.77276 | 22.41534 | 25.20487 | 24.21934 | 23.84806 |
