# Supplementary material for: Identification and Validation of Reference Genes for RT-qPCR Analysis in Non-Heading Chinese Cabbage Flowers
Source: Front Plant Sci. 2016 Jun 10;7:811. doi: 10.3389/fpls.2016.00811 (PMC4901065; doi:10.3389/fpls.2016.00811)
Supplement: Table S2 — Raw Cq values of SRK, Exo70A1 and reference genes under different conditions, as determined by RT-qPCR. SD, different stigma development stages; FT, different floral tissues. [file DataSheet2.docx]

**Table S2 Raw Cq values of *SRK*, *Exo70A1* and reference genes under different conditions, as determined by RT-qPCR. SD: different stigma development stages; FT: different floral tissues.**

|  | SD_I_1 | SD_I_2 | SD_I_3 | SD_I_4 |
| --- | --- | --- | --- | --- |
| *ACT7* | 19.79799 | 20.76208 | 20.13891 | 20.05552 |
|  | 19.65703 | 20.89915 | 20.23441 | 20.26754 |
|  | 19.66218 | 20.70809 | 19.79778 | 20.29293 |
| *EF1α* | 16.43266 | 17.27336 | 16.79235 | 17.10669 |
|  | 16.2894 | 16.90999 | 16.97884 | 17.08976 |
|  | 16.31219 | 17.27936 | 16.97498 | 16.73085 |
| *ACP* | 22.68227 | 23.31882 | 25.89843 | 26.59124 |
|  | 22.62324 | 23.22401 | 25.85207 | 26.50628 |
|  | 22.66312 | 23.35815 | 25.70055 | 26.51469 |
| *SRK* | 32.64762 | 25.76609 | 19.94054 | 20.69128 |
|  | 32.39376 | 25.65189 | 20.00657 | 20.52973 |
|  | 32.18132 | 25.48705 | 20.18344 | 20.70698 |

|  | FT_I_1 | FT_I_2 | FT_I_3 | FT_I_4 | FT_I_5 |
| --- | --- | --- | --- | --- | --- |
| *DNAJ* | 23.92915 | 24.53489 | 24.59203 | 23.60133 | 23.83967 |
|  | 24.03997 | 24.38655 | 24.95209 | 23.64708 | 23.79462 |
|  | 23.8561 | 24.58672 | 24.71754 | 23.41437 | 23.69452 |
| *UKN1* | 23.11098 | 23.37447 | 23.61609 | 22.96496 | 23.14399 |
|  | 23.12665 | 23.40053 | 23.67648 | 22.97804 | 22.78074 |
|  | 23.27982 | 23.29765 | 23.5295 | 23.00716 | 22.85795 |
| *CYP* | 26.03075 | 30.0432 | 29.236 | 27.92855 | 28.20911 |
|  | 26.05983 | 30.07595 | 29.2504 | 27.96613 | 28.28098 |
|  | 25.97417 | 30.28934 | 29.14844 | 28.69968 | 28.0321 |
| *SRK* | 20.76636 | 29.85211 | 21.91133 | 32.37098 | 32.07689 |
|  | 20.64303 | 29.59571 | 21.87953 | 32.42639 | 31.89114 |
|  | 20.74783 | 29.36117 | 21.82787 | 32.12189 | 32.02807 |

|  | FT_I_1 | FT_I_2 | FT_I_3 | FT_I_4 | FT_I_5 | FT_C_1 | FT_C_2 | FT_C_3 | FT_C_4 | FT_C_5 |
| --- | --- | --- | --- | --- | --- | --- | --- | --- | --- | --- |
| *DNAJ* | 23.92915 | 24.53489 | 24.59203 | 23.60133 | 23.83967 | 23.43756 | 23.50011 | 22.93993 | 23.53227 | 22.715821 |
|  | 24.03997 | 24.38655 | 24.95209 | 23.64708 | 23.79462 | 23.55285 | 23.71423 | 22.96434 | 23.56682 | 23.171739 |
|  | 23.8561 | 24.58672 | 24.71754 | 23.41437 | 23.69452 | 23.53608 | 23.70967 | 23.05744 | 22.30292 | 23.124307 |
| *UKN1* | 23.11098 | 23.37447 | 23.61609 | 22.96496 | 23.14399 | 22.54164 | 21.953 | 22.47447 | 22.28861 | 23.125089 |
|  | 23.12665 | 23.40053 | 23.67648 | 22.97804 | 22.78074 | 22.43452 | 21.92071 | 22.45023 | 22.40096 | 23.13837 |
|  | 23.27982 | 23.29765 | 23.5295 | 23.00716 | 22.85795 | 22.46369 | 21.96256 | 22.39817 | 22.60241 | 23.331896 |
| *CYP* | 26.03075 | 30.0432 | 29.236 | 27.92855 | 28.20911 | 22.14653 | 24.34396 | 21.10687 | 24.95469 | 23.168126 |
|  | 26.05983 | 30.07595 | 29.2504 | 27.96613 | 28.28098 | 22.08437 | 24.41149 | 21.05276 | 24.48115 | 23.163859 |
|  | 25.97417 | 30.28934 | 29.14844 | 28.69968 | 28.0321 | 22.08249 | 24.36765 | 21.05408 | 24.53192 | 23.332397 |
| *EXO70A1* | 22.42845 | 23.29992 | 22.33267 | 23.06677 | 22.25434 | 22.49007 | 22.7458 | 22.01667 | 22.77177 | 22.443448 |
|  | 22.16399 | 23.51643 | 22.31567 | 22.2993 | 22.74771 | 22.36279 | 22.75739 | 22.12568 | 22.94998 | 21.971131 |
|  | 22.22531 | 24.59395 | 22.33072 | 22.34772 | 22.62194 | 22.42282 | 22.74471 | 22.04408 | 23.15762 | 22.275384 |
